# Supplementary figures and images for: Hepatic lipase (LIPC) sequencing in individuals with extremely high and low high-density lipoprotein cholesterol levels
Source: PLoS One. 2020 Dec 16;15(12):e0243919. doi: 10.1371/journal.pone.0243919 (PMC7743991; doi:10.1371/journal.pone.0243919)

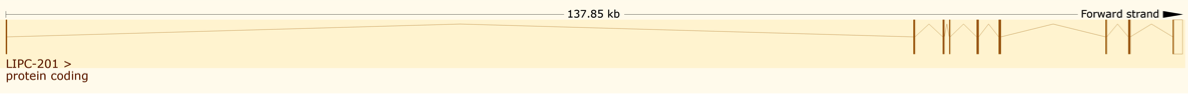

Supplement: S1 Fig — The image was retrieved from http://www.ensembl.org/. Boxes and lines between boxes indicate exons and introns, respectively. Unfilled box indicates untranslated region. (TIF) [file pone.0243919.s001.tif]

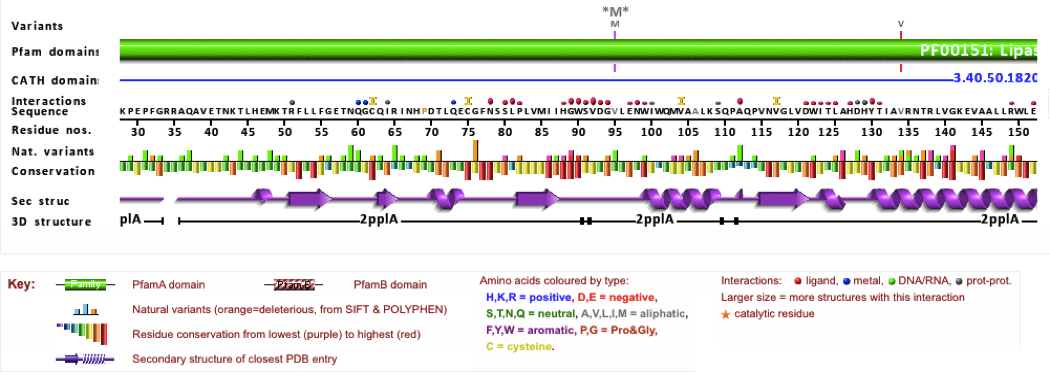

Supplement: S2 Fig — The image was retrieved from the VarSite database (https://www.ebi.ac.uk/thornton-srv/databases/VarSite). (TIF) [file pone.0243919.s002.tif]

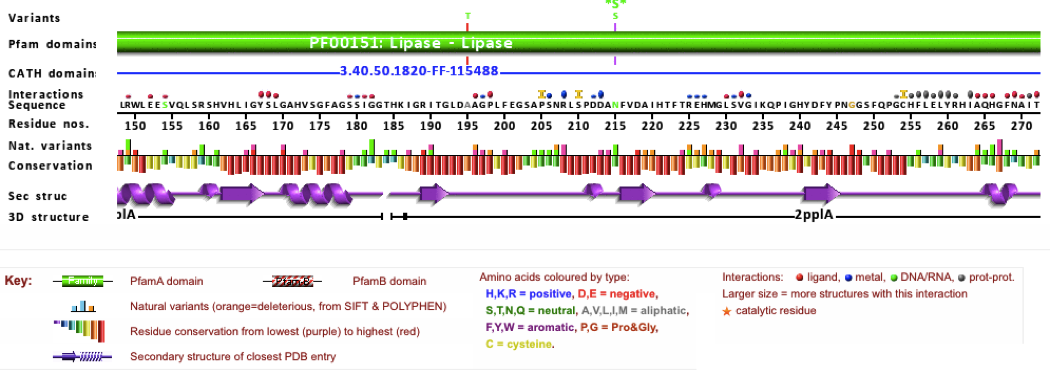

Supplement: S3 Fig — The image was retrieved from the VarSite database (https://www.ebi.ac.uk/thornton-srv/databases/VarSite). (TIF) [file pone.0243919.s003.tif]

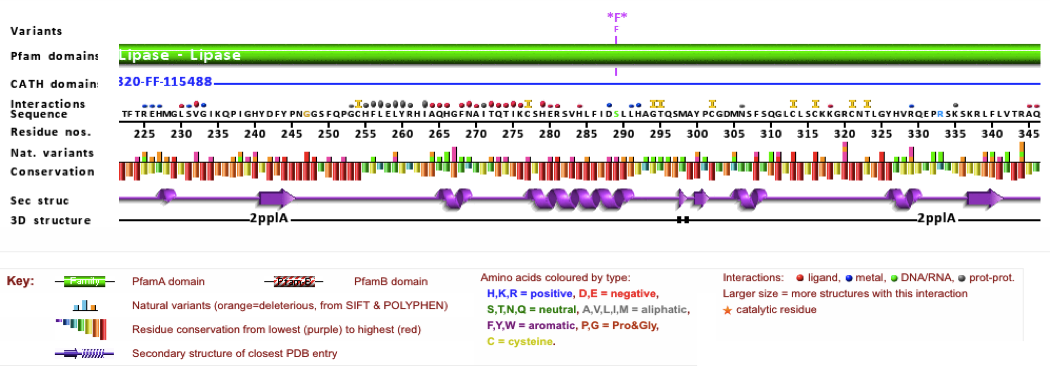

Supplement: S4 Fig — The image was retrieved from the VarSite database (https://www.ebi.ac.uk/thornton-srv/databases/VarSite). (TIF) [file pone.0243919.s004.tif]

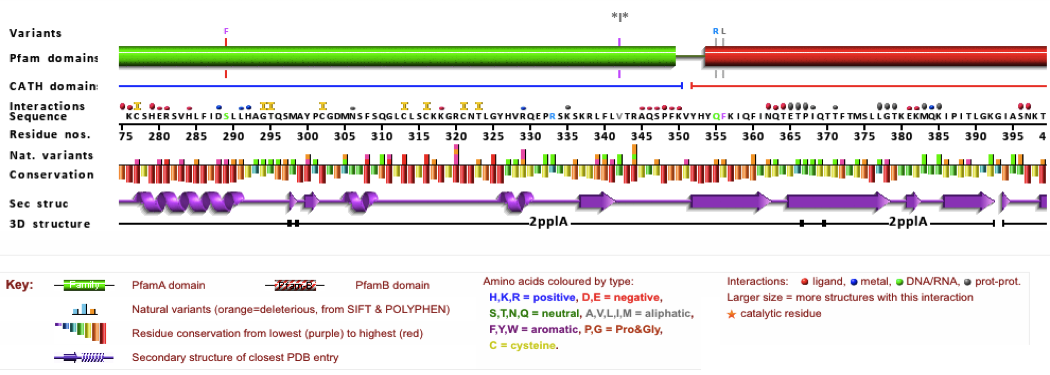

Supplement: S5 Fig — The image was retrieved from the VarSite database (https://www.ebi.ac.uk/thornton-srv/databases/VarSite). (TIF) [file pone.0243919.s005.tif]

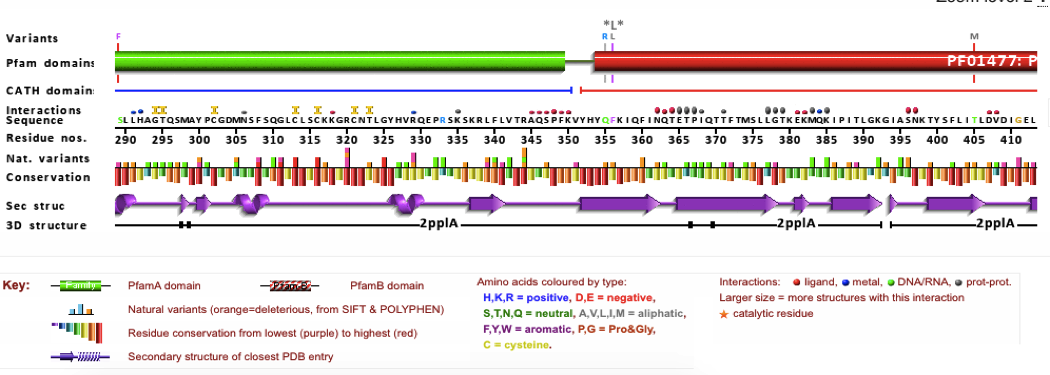

Supplement: S6 Fig — The image was retrieved from the VarSite database (https://www.ebi.ac.uk/thornton-srv/databases/VarSite). (TIF) [file pone.0243919.s006.tif]

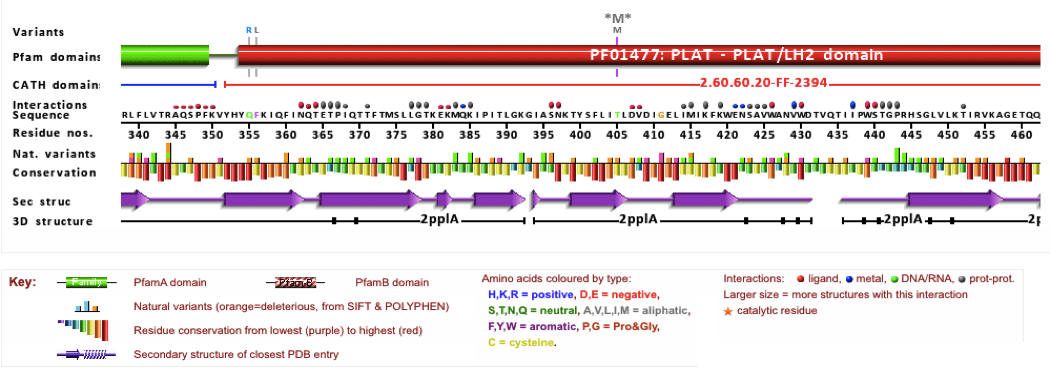

Supplement: S7 Fig — The image was retrieved from the VarSite database (https://www.ebi.ac.uk/thornton-srv/databases/VarSite). (TIF) [file pone.0243919.s007.tif]
