# Supplementary material for: Hepatic lipase (LIPC) sequencing in individuals with extremely high and low high-density lipoprotein cholesterol levels
Source: PLoS One. 2020 Dec 16;15(12):e0243919. doi: 10.1371/journal.pone.0243919 (PMC7743991; doi:10.1371/journal.pone.0243919)
Supplement: S1 Table — (DOCX) [file pone.0243919.s008.docx]

**S1 Table. Primers used in DNA sequencing and PCR**

| **Fragment #** | **Forward Primer Sequence (5’-3’)** | **Reverse Primer Sequence (3’-5’)** | **Fragment #** | **Forward Primer Sequence (5’-3’)** | **Reverse Primer Sequence (3’-5’)** |
| --- | --- | --- | --- | --- | --- |
| Frag-1 | GCTGCAATTTGAAACACCAC | GCTAGCTGCAGGCACTCTTA | Frag-24 | ATTGTGTGAGCCCAGGATTT | TATATCGCCTGCTGTTTTCG |
| Frag-2 | CTGGTCACTGGGATAACAGG | GCAGAGCAGTGAACTGGAAG | Frag-25 | GATAGCCCAGGATGGATGTC | TCTCGGAACCACACTACTGC |
| Frag-3 | ACACAGGGGGACATAGGAAG | GAGGACCTGTTTGGGACTGT | Frag-26 | GCAGGGCATCATTATTTAGC | TTATCTTCCCTCCCACATCA |
| Frag-4 | GAGGAAAATGAACCCTTCCA | GGAGAGGAGGAAAGTTGGTG | Frag-27 | CATGTTTGGGACAATGAACTG | ATCACGAGGTCAACAGATCG |
| Frag-5 | CCATGCACATAGAAGGCACT | CACCCCTAAACCCATAGCAC | Frag-28 | CATGCATACTGCCATTATATGC | AGAGGCTGTGGGTGAGAAGT |
| Frag-6 | TCTCAAACAAGGCACACACA | TCTCAGAGGAAGGGAAAGGA | Frag-29 | TTGACCTTGTGATCCACCTG | TTCTACCGGAGGTTGGTAGC |
| Frag-7 | CACGACCACTACACCATCG | GGCAGGCAATAGGAATTGAG | Frag-30 | CCCAAGAGATATCCCCACTC | TGGCTGATTACTGCTTGGAC |
| Frag-8 | ATCCAACAAGGACCTGCTTT | CTCTTTGTTTTGGCCTGTGA | Frag-31 | GAGAGCTCAGGAGGTCAAGG | GGTGAGGGTCTCCTTTCGTA |
| Frag-9 | TAGGCTTCAACGACACAAGG | CAGTTGGATGGAGAGGGTTC | Frag-32 | ATTTCTTGGCTGGAGCTCTG | GTAGCCTGACCACCTTGGAT |
| Frag-10 | GACAGCTCATTTCCTCATGC | GCGGGACATTTCACTGTATG | Frag-33 | CAAGAGTCAGGCCAGCTACA | AGGGCATTCCAAAGTTCAAG |
| Frag-11 | CCAAGTAGGGAAGGAAACCA | TAGAAAACCTCGCCGTAACC | Frag-34 | GAGTCAGGCTGGAATTTGGT | AGGTGGGTAACAGGGTGAAG |
| Frag-12 | GCCTCAGGCATCATAACCTT | CATCGAGAAGCCACAACCTA | Frag-35 | AGACAGGGAAGCCAGAGATG | TTCTAGGAAGAAGCCGCTGT |
| Frag-13 | ACAGACATATTGCCCAGCAC | AGATGATGCGGAGTGTGAAG | Frag-36 | AAATCCACTGCTGCCTCTG | AATCCTTGTCCTGGCACCT |
| Frag-14 | CCACCCAACAAAGCTCAGTA | ATTTAAAATGCCCAGGTGCT | Frag-37 | GTGGGGAGCAAAGAAAAGG | GGCCAAAGAGTCATGTGAGA |
| Frag-15 | GGTTACTGGGCAATGTCCTT | GACTTCTGAGCCCTTTGGAC | Frag-38 | AGATGTATGCCCCTGAGTCC | TTCCTGGTTAGGTCCTGGAG |
| Frag-16 | AACACGCTGGGCTACCAC | CCCTGTTCCAGGTCTCCTTA | Frag-39 | AGATGTATGCCCCTGAGTCC | TTCCTGGTTAGGTCCTGGAG |
| Frag-17 | TCTCACTGTTTCATCGCACA | CCTCTTTTCTTCCCAGGTGA | Frag-40 | TGCAGTGCTGGACATTAACA | TCACCATCTCCACAACCCTA |
| Frag-18 | TCATGAGGAGCAGTTCAACC | GAACTGGGGAGAATTTGTGG | Frag-41 | GCAGACCAAAATCCCAGAAT | TGCACAGATACTCCAGAAAGATG |
| Frag-19 | TCCACCTGCAGGGTTCAA | TGAAATGAGAGGAGGCACTG | Frag-42 | TGGAATGTCATGAAGCAGGT | TGACCACATTTGCCTTGACT |
| Frag-20 | TAAACATCGCCTCCTGATTG | CCCTGCTTCTTCTGGAACTC | Frag-43 | CTTGACCTTAGACGCAATTCA | TTGGGCGAAGTAGTAGGTCA |
| Frag-21 | GCACTTGCTTCTGCTGCTC | TTGTAGAGATGGCGTCTTGC | Frag-44 | TTAGGAGGAAGGGTTGAGGA | TGGCTCTTGCATTTGTACTT |
| Frag-22 | CCAGTCAGCACAACAGTCCT | GGATTGTAACCCTGTGTGGA | Frag-45 | AATGCTGTGTTTGCTTCCTG | GCAAATGCTTTGAGACATTCC |
| Frag-23 | AAGCCCTCTTTGATGTTTGC | CTTGAGTTTGCAGGAGCACT | Frag-46 | TCCCACATGTCCTTGCTAGT | GTGAGGGTCAACTTCCTGGT |
