# Supplementary material for: Hepatic lipase (LIPC) sequencing in individuals with extremely high and low high-density lipoprotein cholesterol levels
Source: PLoS One. 2020 Dec 16;15(12):e0243919. doi: 10.1371/journal.pone.0243919 (PMC7743991; doi:10.1371/journal.pone.0243919)
Supplement: S2 Table — (DOCX) [file pone.0243919.s009.docx]

**S2 Table. Sequencing results for the *LIPC* gene in NHWs (n=95)**

| Ref SNP ID | Alleles | Location | Call rate | HW-P | High HDL-C MAF | Low HDL-C  MAF | Total MAF | *P*-value | RegulomeDB scores | Related regulatory elements | rSNP |
| --- | --- | --- | --- | --- | --- | --- | --- | --- | --- | --- | --- |
| rs2070895 | G>A | 5' Flanking | 97.9 | 0.9604 | 0.207 | 0.128 | 0.167 | 0.149 | 2b |  |  |
| rs8192701 | C>T | Intron 1 | 97.9 | 0.5447 | 0.098 | 0.117 | 0.108 | 0.6727 | 4 | lncRNA region | rSNP |
| rs11631342 | A>G | Intron 1 | 97.9 | 1 | 0.022 | 0 | 0.011 | 0.1506 | 4 | lncRNA region | rSNP |
| rs11633191 | T>C | Intron 2 | 98.9 | 0.844 | 0.149 | 0.17 | 0.16 | 0.6904 | 4 |  |  |
| rs2233741 | A>T | Intron 2 | 97.9 | 1 | 0.141 | 0.074 | 0.108 | 0.1413 | 2b |  |  |
| rs2233739 | C>A | Intron 2 | 97.9 | 0.4612 | 0.098 | 0.106 | 0.102 | 0.8472 | 4 |  |  |
| rs2233738 | A>G | Intron 2 | 97.9 | 1 | 0 | 0.011 | 0.005 | 0.3212 | 4 |  |  |
| rs79783761 | C>A | Intron 2 | 96.8 | 1 | 0.067 | 0.043 | 0.054 | 0.4708 | 3a |  |  |
| rs78415410 | G>C | Intron 2 | 97.9 | 1 | 0.011 | 0 | 0.005 | 0.3004 | 2b |  |  |
| rs11631482 | T>A | Intron 2 | 97.9 | 1 | 0.1 | 0.073 | 0.086 | 0.5103 | 5 |  |  |
| rs866994189* | T>A | Intron 2 | 97.9 | 1 | 0 | 0.01 | 0.005 | 0.3316 | 5 |  |  |
| rs12909325 | G>A | Intron 2 | 96.8 | 0.8176 | 0.422 | 0.489 | 0.457 | 0.3607 | 5 | circRNA region | rSNP |
| rs59699190 | T>C | Intron 2 | 96.8 | 1 | 0.122 | 0.138 | 0.13 | 0.7462 | 5 | circRNA region | rSNP |
| rs6076 | G>A | Intron 2 | 96.8 | 0.5538 | 0.1 | 0.117 | 0.109 | 0.7108 | 5 | circRNA region | rSNP |
| rs6078 | G>A | Exon 3 | 96.8 | 1 | 0.011 | 0 | 0.005 | 0.3055 | 5 |  |  |
| [rs553734055](https://www.ncbi.nlm.nih.gov/projects/SNP/snp_ref.cgi?rs=553734055) | del2>ins2 | Intron 3 | 94.7 | 1 | 0 | 0.011 | 0.006 | 0.3375 | 5 |  |  |
| rs2242061 | T>C | Intron 3 | 94.7 | 0.7966 | 0.221 | 0.213 | 0.217 | 0.8943 | 5 |  |  |
| rs2242062 | A>G | Intron 3 | 94.7 | 0.9633 | 0.093 | 0.085 | 0.089 | 0.8521 | 5 |  |  |
| [rs762790338](https://www.ncbi.nlm.nih.gov/projects/SNP/snp_ref.cgi?rs=762790338) | A>G | Intron 3 | 94.7 | 1 | 0.012 | 0 | 0.006 | 0.2945 | 5 |  |  |
| rs690 | T>G | Exon 4 | 94.7 | 0.3522 | 0.5 | 0.362 | 0.428 | 0.061 | 5 |  |  |
| rs16940472 | C>A | Intron 4 | 100 | 0.73 | 0.106 | 0.188 | 0.147 | 0.1148 | 2b |  |  |
| [rs534846447](https://www.ncbi.nlm.nih.gov/projects/SNP/snp_ref.cgi?rs=rs534846447) | G>A | Intron 4 | 100 | 1 | 0 | 0.01 | 0.005 | 0.3211 | 4 |  |  |
| rs4572327 | T>A | Intron 4 | 100 | 1 | 0.021 | 0.031 | 0.026 | 0.6676 | 4 |  |  |
| rs41292506 | T>C | Intron 4 | 100 | 1 | 0.032 | 0.062 | 0.047 | 0.3211 | 4 |  |  |
| rs12593954 | T>C | Intron 4 | 95.8 | 1 | 0.022 | 0.033 | 0.027 | 0.6323 | 5 |  |  |
| rs11852861 | C>T | Intron 4 | 95.8 | 1 | 0.446 | 0.378 | 0.412 | 0.3523 | 5 |  |  |
| rs553424005 | C>G | Intron 4 | 92.6 | 1 | 0.011 | 0.011 | 0.011 | 1 | 5 |  |  |
| rs62002609 | G>A | Intron 4 | 92.6 | 1 | 0.034 | 0.011 | 0.023 | 0.3117 | 5 |  |  |
| rs56143289 | T>C | Intron 4 | 92.6 | 0.2488 | 0.432 | 0.455 | 0.443 | 0.7615 | 5 |  |  |
| rs12592139 | G>A | Intron 4 | 92.6 | 1 | 0.023 | 0.034 | 0.028 | 0.65 | 5 |  |  |
| rs74017973 | G>T | Intron 4 | 97.9 | 1 | 0.065 | 0.053 | 0.059 | 0.7281 | 5 |  |  |
| rs12592127 | A>G | Intron 4 | 97.9 | 0.6302 | 0.065 | 0.064 | 0.065 | 0.9693 | 5 |  |  |
| rs866980505* | G>A | Intron 4 | 92.6 | 1 | 0.011 | 0 | 0.006 | 0.3159 | 3a |  |  |
| rs17190650 | C>T | Intron 4 | 92.6 | 0.873 | 0.511 | 0.489 | 0.5 | 0.763 | 3a |  |  |
| rs11632970 | G>C | Intron 4 | 89.5 | 1 | 0.146 | 0.227 | 0.188 | 0.1774 | 4 |  |  |
| rs55733523 | C>T | Intron 4 | 95.8 | 0.3878 | 0.2 | 0.13 | 0.165 | 0.206 | 4 |  |  |
| rs11633043 | G>A | Intron 4 | 94.7 | 1 | 0.091 | 0.163 | 0.128 | 0.1473 | 2b |  |  |
| rs4775075 | A>G | Intron 4 | 97.9 | 0.7247 | 0.076 | 0.064 | 0.07 | 0.7431 | 4 |  |  |
| rs6080 | C>A | Intron 4 | 97.9 | 1 | 0.043 | 0.053 | 0.048 | 0.7576 | 4 |  |  |
| rs6082 | A>G | Exon 5 | 97.9 | 0.7247 | 0.076 | 0.064 | 0.07 | 0.7431 | 4 |  |  |
| rs6083 | A>G | Exon 5 | 97.9 | 0.8433 | 0.38 | 0.34 | 0.36 | 0.5698 | 4 |  |  |
| rs6084 | G>C | Exon 5 | 97.9 | 0.5996 | 0.424 | 0.5 | 0.462 | 0.2981 | 4 |  |  |
| rs1973023 | C>T | Intron 5 | 98.9 | 0.5771 | 0.255 | 0.191 | 0.223 | 0.2935 | 4 |  |  |
| rs1973024 | C>T | Intron 5 | 100 | 0.5569 | 0.255 | 0.188 | 0.221 | 0.26 | 4 |  |  |
| rs2242063 | G>A | Intron 5 | 97.9 | 1 | 0.167 | 0.115 | 0.14 | 0.306 | 4 |  |  |
| rs10459627 | C>T | Intron 5 | 96.8 | 1 | 0.556 | 0.489 | 0.478 | 0.3689 | 4 |  |  |
| rs2242064 | G>T | Intron 5 | 94.7 | 0.2349 | 0.256 | 0.372 | 0.317 | 0.0932 | 4 |  |  |
| rs2242065 | C>T | Intron 5 | 93.7 | 0.8505 | 0.081 | 0.076 | 0.079 | 0.8954 | 2a |  |  |
| rs36205683 | C>T | Intron 5 | 93.7 | 1 | 0.012 | 0.011 | 0.011 | 0.9617 | 2b |  |  |
| rs2242066 | G>A | Intron 5 | 93.7 | 1 | 0.163 | 0.12 | 0.14 | 0.4069 | 3a |  |  |
| rs549130875 | C>T | Intron 5 | 94.7 | 1 | 0.011 | 0.011 | 0.011 | 0.9748 | 4 |  |  |
| [rs756160225](https://www.ncbi.nlm.nih.gov/projects/SNP/snp_ref.cgi?rs=rs756160225) | C>T | Intron 5 | 94.7 | 1 | 0.011 | 0 | 0.006 | 0.3052 | 4 |  |  |
| rs143731122 | ins5>del5 | Intron 5 | 96.8 | 0.1575 | 0.326 | 0.185 | 0.255 | 0.028 | 4 |  |  |
| rs7165654 | G>A | Intron 5 | 98.9 | 0.6751 | 0.266 | 0.191 | 0.229 | 0.2242 | 4 |  |  |
| rs12913969 | G>A | Intron 5 | 98.9 | 1 | 0.191 | 0.117 | 0.154 | 0.1575 | 4 |  |  |
| rs121912502 | C>T | Exon 6 | 97.9 | 1 | 0.011 | 0 | 0.005 | 0.3108 | 4 |  |  |
| rs56176178 | C>G | Intron 6 | 98.9 | 0.5993 | 0.207 | 0.146 | 0.176 | 0.2742 | 4 |  |  |
| rs188556987 | T>C | Intron 6 | 98.9 | 1 | 0.011 | 0 | 0.005 | 0.3057 | 2b |  |  |
| rs7166788 | C>G | Intron 6 | 100 | 0.7555 | 0.447 | 0.479 | 0.463 | 0.6547 | 4 |  |  |
| [rs548590424](https://www.ncbi.nlm.nih.gov/projects/SNP/snp_ref.cgi?rs=rs548590424) | C>G | Intron 6 | 95.8 | 1 | 0.011 | 0 | 0.005 | 0.3107 | 5 |  |  |
| [rs575452965](https://www.ncbi.nlm.nih.gov/projects/SNP/snp_ref.cgi?rs=rs575452965) | A>G | Intron 6 | 95.8 | 1 | 0.011 | 0 | 0.005 | 0.3107 | 5 |  |  |
| rs867460379* | T>G | Intron 6 | 95.8 | 1 | 0.011 | 0 | 0.005 | 0.3107 | 5 |  |  |
| rs1007542 | G>A | Intron 6 | 97.9 | 1 | 0 | 0.01 | 0.005 | 0.3316 | 5 |  |  |
| rs11632627 | A>G | Intron 6 | 97.9 | 0.9121 | 0.189 | 0.292 | 0.242 | 0.1019 | 5 |  |  |
| rs56171273 | A>G | Intron 6 | 51.6 | 0.0004 | 0.326 | 0.346 | 0.337 | 0.8338 | 6 |  |  |
| rs67727760 | T>C | Intron 6 | 55.8 | 0.0002 | 0.333 | 0.345 | 0.34 | 0.901 | 5 |  |  |
| rs56122478 | del8>ins8 | Intron 6 | 81.1 | 1 | 0.192 | 0.145 | 0.169 | 0.4308 | 5 |  |  |
| rs78861621 | C>T | Intron 6 | 71.6 | 0.0133 | 0.155 | 0.103 | 0.125 | 0.3589 | 5 |  |  |
| rs74837693 | C>T | Intron 6 | 96.8 | 1 | 0.011 | 0.021 | 0.016 | 0.5862 | 2b |  |  |
| [rs866428151](https://www.ncbi.nlm.nih.gov/projects/SNP/snp_ref.cgi?rs=866428151)* | A>G | Intron 6 | 96.8 | 1 | 0 | 0.011 | 0.005 | 0.3265 | 4 |  |  |
| [rs867178473](https://www.ncbi.nlm.nih.gov/projects/SNP/snp_ref.cgi?rs=867178473)* | C>A | Intron 6 | 97.9 | 1 | 0.011 | 0 | 0.005 | 0.3108 | 4 |  |  |
| rs871804 | C>T | Intron 6 | 98.9 | 1 | 0.185 | 0.115 | 0.149 | 0.1765 | 4 |  |  |
| rs143861207 | ins2>del2 | Intron 6 | 97.9 | 1 | 0 | 0.043 | 0.022 | 0.0455 | 4 |  |  |
| rs118174606 | A>G | Intron 6 | 96.8 | 1 | 0 | 0.033 | 0.016 | 0.0808 | 4 |  |  |
| rs12908645 | T>C | Intron 6 | 95.8 | 1 | 0.167 | 0.12 | 0.143 | 0.3639 | 3a |  |  |
| rs45500398 | T>A | Intron 6 | 95.8 | 1 | 0.522 | 0.467 | 0.495 | 0.4595 | 5 |  |  |
| rs12595265 | G>C | Intron 6 | 97.9 | 0.8205 | 0.076 | 0.074 | 0.075 | 0.9666 | 5 |  |  |
| rs11071389 | G>T | Intron 6 | 97.9 | 0.4195 | 0.239 | 0.191 | 0.215 | 0.4291 | 5 |  |  |
| rs28524122 | G>A | Intron 6 | 100 | 1 | 0.17 | 0.115 | 0.142 | 0.2722 | 5 |  |  |
| rs72743035 | G>C | Intron 6 | 100 | 1 | 0.053 | 0.052 | 0.053 | 0.9727 | 5 |  |  |
| rs7178362 | T>C | Intron 6 | 100 | 1 | 0.106 | 0.146 | 0.126 | 0.4131 | 5 |  |  |
| rs17190678 | G>C | Intron 6 | 100 | 0.5924 | 0.436 | 0.479 | 0.458 | 0.552 | 5 |  |  |
| rs144681617 | C>T | Intron 6 | 100 | 1 | 0.011 | 0.021 | 0.016 | 0.573 | 4 |  |  |
| rs147941779 | G>A | Intron 6 | 100 | 1 | 0 | 0.021 | 0.011 | 0.1595 | 3a |  |  |
| rs41294813 | C>T | Intron 6 | 100 | 0.7555 | 0.447 | 0.479 | 0.463 | 0.6547 | 3a |  |  |
| rs78312967 | G>A | Intron 6 | 100 | 0.8063 | 0.074 | 0.073 | 0.074 | 0.9674 | 4 |  |  |
| rs56010348 | G>T | Intron 6 | 100 | 0.7555 | 0.447 | 0.479 | 0.463 | 0.6547 | 2c |  |  |
| rs867569005* | A>G | Intron 6 | 100 | 1 | 0 | 0.01 | 0.005 | 0.3211 | 2b |  |  |
| rs138700694 | G>A | Intron 6 | 100 | 1 | 0.011 | 0.01 | 0.011 | 0.9881 | 2b |  |  |
| rs6494018 | T>G | Intron 6 | 98.9 | 1 | 0.213 | 0.298 | 0.255 | 0.1809 | 2b |  |  |
| rs35631005 | C>T | Intron 6 | 97.9 | 1 | 0 | 0.011 | 0.005 | 0.3108 | 4 |  |  |
| rs866218418* | T>G | Intron 6 | 97.9 | 1 | 0.011 | 0 | 0.005 | 0.3212 | 3a |  |  |
| rs35892254 | A>G | Intron 6 | 97.9 | 0.9508 | 0.478 | 0.489 | 0.484 | 0.8796 | 5 |  |  |
| rs866651605* | G>A | Intron 6 | 97.9 | 1 | 0.011 | 0 | 0.005 | 0.3108 | 5 |  |  |
| rs72743041 | T>C | Intron 6 | 91.6 | 1.9982E-08 | 0.378 | 0.467 | 0.425 | 0.2341 | NA |  |  |
| rs868799105* | A>G | Intron 6 | 91.6 | 1 | 0.012 | 0 | 0.006 | 0.2881 | 5 |  |  |
| rs866549972* | C>G | Intron 6 | 91.6 | 1 | 0.012 | 0 | 0.006 | 0.2881 | 5 |  |  |
| rs67262567 | Ins1>del1 | Intron 6 | 91.6 | 0.603 | 0.134 | 0.098 | 0.115 | 0.4534 | 7 |  |  |
| rs2414593 | C>T | Intron 6 | 100 | 1 | 0 | 0.01 | 0.005 | 0.3211 | 5 |  |  |
| rs866663393* | G>A | Intron 6 | 98.9 | 1 | 0.011 | 0 | 0.005 | 0.3057 | 5 |  |  |
| rs2414594 | G>A | Intron 6 | 100 | 1 | 0 | 0.01 | 0.005 | 0.3211 | 5 |  |  |
| rs61207362* | Ins30>del30 | Intron 6 | 100 | 1 | 0 | 0.01 | 0.005 | 0.3211 | 5 |  |  |
| rs2414595* | C>A | Intron 6 | 100 | 1 | 0 | 0.01 | 0.005 | 0.3211 | 5 |  |  |
| rs2414598 | A>G | Intron 6 | 97.9 | 1 | 0 | 0.011 | 0.005 | 0.3108 | 4 |  |  |
| rs868769860* | T>G | Intron 6 | 97.9 | 1 | 0 | 0.011 | 0.005 | 0.3108 | 4 |  |  |
| rs2414599 | T>C | Intron 6 | 97.9 | 1 | 0 | 0.011 | 0.005 | 0.3108 | 4 |  |  |
| rs2414600 | A>C | Intron 6 | 97.9 | 1 | 0 | 0.011 | 0.005 | 0.3108 | 3a |  |  |
| rs2414601 | T>C | Intron 6 | 97.9 | 1 | 0 | 0.011 | 0.005 | 0.3108 | 3a |  |  |
| rs28455962 | T>A | Intron 6 | 98.9 | 1 | 0 | 0.011 | 0.005 | 0.316 | 5 |  |  |
| rs4444272 | C>A | Intron 6 | 95.8 | 1 | 0 | 0.011 | 0.005 | 0.3107 | 3a |  |  |
| rs4528512 | A>T | Intron 6 | 95.8 | 1 | 0.435 | 0.444 | 0.44 | 0.8955 | 3a |  |  |
| rs11858020 | T>A | Intron 6 | 95.8 | 1 | 0.446 | 0.456 | 0.451 | 0.8932 | 4 |  |  |
| rs7179747 | C>A | Intron 6 | 93.7 | 1 | 0 | 0.011 | 0.006 | 0.3105 | 2b |  |  |
| rs7179940 | G>C | Intron 6 | 93.7 | 1 | 0 | 0.011 | 0.006 | 0.3105 | 4 |  |  |
| rs7181367 | C>T | Intron 6 | 93.7 | 1 | 0 | 0.011 | 0.006 | 0.3105 | 2b |  |  |
| rs7179938 | C>A | Intron 6 | 93.7 | 1 | 0 | 0.011 | 0.006 | 0.3105 | 3a |  |  |
| rs11639204 | A>G | Intron 6 | 93.7 | 1 | 0 | 0.011 | 0.006 | 0.3105 | 2a |  |  |
| rs7180130 | G>A | Intron 6 | 93.7 | 1 | 0 | 0.011 | 0.006 | 0.3105 | 4 |  |  |
| rs7181592 | C>T | Intron 6 | 97.9 | 1 | 0 | 0.01 | 0.005 | 0.3316 | 2b |  |  |
| rs7180795 | A>G | Intron 6 | 97.9 | 1 | 0 | 0.01 | 0.005 | 0.3316 | 5 |  |  |
| rs7181945 | G>T | Intron 6 | 97.9 | 1 | 0 | 0.01 | 0.005 | 0.3316 | 5 |  |  |
| rs33931419 | C>G | Intron 6 | 97.9 | 0.1947 | 0.326 | 0.436 | 0.382 | 0.1223 | 7 |  |  |
| rs527382659 | A>C | Intron 6 | 97.9 | 1 | 0.011 | 0 | 0.005 | 0.3108 | 6 |  |  |
| rs77010273 | T>C | Intron 7 | 97.9 | 0.1106 | 0.087 | 0.053 | 0.07 | 0.3665 | 2b | circRNA region | rSNP |
| rs72062747 | del2>ins2 | Intron 7 | 98.9 | 1 | 0 | 0.01 | 0.005 | 0.3263 | 2b |  |  |
| rs4774305 | G>C | Intron 7 | 97.9 | 1 | 0 | 0.01 | 0.005 | 0.3316 | 4 | circRNA region | rSNP |
| rs1869129 | T>C | Intron 7 | 100 | 1 | 0 | 0.01 | 0.005 | 0.3211 | 4 | circRNA region | rSNP |
| rs1869130 | C>T | Intron 7 | 100 | 1 | 0 | 0.01 | 0.005 | 0.3211 | 3a | circRNA region | rSNP |
| rs12438032 | G>A | Intron 7 | 100 | 1 | 0 | 0.01 | 0.005 | 0.3211 | 4 | circRNA region | rSNP |
| rs34964641 | T>G | Intron 7 | 100 | 1 | 0 | 0.01 | 0.005 | 0.3211 | 5 | circRNA region | rSNP |
| rs35925692 | ins1>del1 | Intron 7 | 100 | 1 | 0 | 0.01 | 0.005 | 0.3211 | 5 | circRNA region | rSNP |
| rs1869131 | A>T | Intron 7 | 100 | 1 | 0 | 0.01 | 0.005 | 0.3211 | 5 | circRNA region | rSNP |
| rs4775079 | C>T | Intron 7 | 100 | 1 | 0 | 0.01 | 0.005 | 0.3211 | 5 | circRNA region | rSNP |
| rs1869132 | G>T | Intron 7 | 100 | 0.9916 | 0.394 | 0.417 | 0.405 | 0.7463 | 5 | circRNA region | rSNP |
| rs865868148* | T>C | Intron 7 | 100 | 1 | 0.011 | 0 | 0.005 | 0.3109 | 5 |  |  |
| rs8026372 | A>G | Intron 7 | 100 | 1 | 0 | 0.01 | 0.005 | 0.3211 | 5 | circRNA region | rSNP |
| rs1839928 | A>G | Intron 7 | 100 | 1 | 0 | 0.01 | 0.005 | 0.3211 | 5 | circRNA region | rSNP |
| rs1839927 | A>G | Intron 7 | 100 | 1 | 0 | 0.01 | 0.005 | 0.3211 | 5 | circRNA region | rSNP |
| rs8027708 | T>A | Intron 7 | 100 | 0.5676 | 0.436 | 0.417 | 0.426 | 0.7858 | 5 | circRNA region | rSNP |
| rs36017602 | G>A | Intron 7 | 100 | 1 | 0.17 | 0.104 | 0.137 | 0.1854 | 4 | circRNA region | rSNP |
| rs8030893 | C>G | Intron 7 | 100 | 0.5676 | 0.436 | 0.417 | 0.426 | 0.7858 | 4 | circRNA region | rSNP |
| rs8030903 | T>C | Intron 7 | 100 | 1 | 0 | 0.01 | 0.005 | 0.3211 | 4 | circRNA region | rSNP |
| rs10851636 | C>T | Intron 7 | 100 | 1 | 0 | 0.01 | 0.005 | 0.3211 | 4 | circRNA region | rSNP |
| rs10851637 | G>C | Intron 7 | 100 | 0.5676 | 0.436 | 0.417 | 0.426 | 0.7858 | 4 | circRNA region | rSNP |
| rs7170227 | G>A | Intron 7 | 100 | 1 | 0 | 0.01 | 0.005 | 0.3211 | 3a | circRNA region | rSNP |
| rs117911817 | G>A | Intron 7 | 98.9 | 1 | 0.033 | 0 | 0.016 | 0.0745 | 5 | circRNA region | rSNP |
| rs139878091 | A>G | Intron 7 | 98.9 | 1 | 0.011 | 0 | 0.005 | 0.3057 | 5 | circRNA region | rSNP |
| rs17301857 | A>G | Intron 7 | 98.9 | 1 | 0.163 | 0.104 | 0.133 | 0.2346 | 5 | circRNA region | rSNP |
| rs7172821 | T>C | Intron 7 | 98.9 | 1 | 0.054 | 0.094 | 0.074 | 0.3036 | 5 | circRNA region | rSNP |
| rs35412158 | G>A | Intron 7 | 100 | 1 | 0 | 0.01 | 0.005 | 0.3211 | 5 | circRNA region | rSNP |
| rs7171818 | A>G | Intron 7 | 98.9 | 0.1378 | 0.223 | 0.277 | 0.25 | 0.3997 | 5 | circRNA region | rSNP |
|  | A>T |  | 98.9 | 0.2465 | 0.106 | 0.064 | 0.085 | 0.2958 | 5 |  |  |
|  | G>T |  | 98.9 | 0.1084 | 0.053 | 0.085 | 0.069 | 0.3885 | 5 |  |  |
| rs17301864 | C>T | Intron 7 | 100 | 1 | 0.17 | 0.104 | 0.137 | 0.1854 | 5 | circRNA region | rSNP |
| rs11071390 | A>G | Intron 7 | 95.8 | 1 | 0 | 0.011 | 0.005 | 0.3319 | 5 | circRNA region | rSNP |
| rs144831345 | G>A | Intron 7 | 95.8 | 1 | 0 | 0.011 | 0.005 | 0.3319 | 5 | circRNA region | rSNP |
| rs143186931 | A>G | Intron 7 | 95.8 | 1 | 0.023 | 0 | 0.011 | 0.1416 | 5 | circRNA region | rSNP |
| rs117852639 | C>A | Intron 7 | 95.8 | 1 | 0 | 0.021 | 0.011 | 0.1688 | 5 | circRNA region | rSNP |
| rs113298164 | C>T | Exon 8 | 96.8 | 1 | 0.011 | 0 | 0.005 | 0.3055 | 5 | circRNA region | rSNP |
| rs6077 | C>T | Intron 8 | 100 | 1 | 0 | 0.01 | 0.005 | 0.3211 | 5 |  |  |
| rs3751542 | T>C | Intron 8 | 100 | 0.8508 | 0.277 | 0.312 | 0.295 | 0.5873 | 5 |  |  |
| rs67897154 | A>G | Intron 8 | 64.2 | 0.0113 | 0.293 | 0.219 | 0.254 | 0.3462 | 5 |  |  |
| rs115464904 | C>T | Intron 8 | 100 | 1 | 0.011 | 0 | 0.005 | 0.3109 | 4 |  |  |
| rs17269397 | G>A | Intron 8 | 100 | 0.3214 | 0.426 | 0.427 | 0.426 | 0.9828 | 4 |  |  |
| rs867986131* | T>C | Intron 8 | 84.2 | 1 | 0 | 0.013 | 0.006 | 0.2916 | 3a |  |  |
| rs3829460 | T>A | Intron 8 | 72.6 | 0.288 | 0.258 | 0.319 | 0.29 | 0.4236 | 5 |  |  |
| rs10152558 | C>T | Intron 8 | 14.7 | 0.742 | 0.417 | 0.125 | 0.25 | 0.0778 | 6 |  |  |
| rs28602186 | G>A | Intron 8 | 94.7 | 0.8586 | 0.156 | 0.1 | 0.128 | 0.2643 | 4 |  |  |
| rs7175421 | T>C | Intron 8 | 97.9 | 0.9202 | 0.289 | 0.312 | 0.301 | 0.7257 | 5 |  |  |
| rs111285504 | G>A | Intron 8 | 97.9 | 1 | 0.011 | 0 | 0.005 | 0.3004 | 7 |  |  |
| rs28515698 | T>C | Intron 8 | 62.1 | 0.5404 | 0.136 | 0.135 | 0.136 | 0.978 | 6 |  |  |
| rs4562992 | A>C | Intron 8 | 61.1 | 0.4034 | 0.281 | 0.385 | 0.328 | 0.2381 | 7 |  |  |
| rs28458188 | T>C | Intron 8 | 77.9 | 0.985 | 0.171 | 0.128 | 0.149 | 0.4605 | 7 |  |  |
| rs147008875 | G>C | Intron 8 | 98.9 | 1 | 0.011 | 0.01 | 0.011 | 0.9759 | 4 |  |  |
| rs112520439 | T>C | Intron 8 | 98.9 | 1 | 0.011 | 0.01 | 0.011 | 0.9759 | 4 |  |  |
| rs67688669 | C>T | Intron 8 | 93.7 | 0.836 | 0.42 | 0.378 | 0.399 | 0.561 | 2b |  |  |
| rs868706856* | G>A | Intron 8 | 98.9 | 1 | 0 | 0.01 | 0.005 | 0.3263 | 3a |  |  |
| rs6074 | C>A | Exon 9 | 98.9 | 1 | 0.174 | 0.104 | 0.138 | 0.1661 | 3a |  |  |
| rs28427123 | T>C | 3' Flanking | 96.8 | 0.9318 | 0.159 | 0.104 | 0.13 | 0.2691 | 3a |  |  |
| rs1978578 | T>C | 3' Flanking | 96.8 | 0.8461 | 0.318 | 0.406 | 0.364 | 0.2149 | 3a |  |  |
| HW-P: Hardy-Weinberg equilibrium p-value, MAF: Minor allele frequency, *Novel variants: Submitted to the dbSNP database as a first submission by handle ID: Kamboh  RegulomeDB scores were generated by using <http://regulome.stanford.edu/>. Scores represents; 1a- eQTL + TF binding + matched TF motif + matched DNase Footprint + DNase peak; 1b- eQTL + TF binding + any motif + DNase Footprint + DNase peak; 1c- eQTL + TF binding + matched TF motif + DNase peak; 1d- eQTL + TF binding + any motif + DNase peak; 1e- eQTL + TF binding + matched TF motif; 1f- eQTL + TF binding / DNase peak; 2a- TF binding + matched TF motif + matched DNase Footprint + DNase peak; 2b- TF binding + any motif + DNase Footprint + DNase peak; 2c- TF binding + matched TF motif + DNase peak; 3a- TF binding + any motif + DNase peak; 3b- TF binding + matched TF motif; 4- TF binding + DNase peak; 5-TF binding or DNase peak; 6-other. | | | | | | | | | | | |
