# Supplementary material for: Hepatic lipase (LIPC) sequencing in individuals with extremely high and low high-density lipoprotein cholesterol levels
Source: PLoS One. 2020 Dec 16;15(12):e0243919. doi: 10.1371/journal.pone.0243919 (PMC7743991; doi:10.1371/journal.pone.0243919)
Supplement: S3 Table — (DOCX) [file pone.0243919.s010.docx]

**S3 Table. Sequencing results for the *LIPC* gene in African blacks (n=95)**

| Ref SNP ID | Alleles | Location | Call rate | HW-P | Low HDL-C MAF | High HDL-C MAF | Total MAF | *P-*value | Regulome scores | Related regulatory elements | rSNP |
| --- | --- | --- | --- | --- | --- | --- | --- | --- | --- | --- | --- |
| rs2070895 | A>G | 5' flanking | 100 | 0.2683 | 0.457 | 0.344 | 0.4 | 0.1097 | 2b |  |  |
| rs150474806 | G>C | 5' flanking | 100 | 1 | 0.011 | 0.01 | 0.011 | 0.9881 | 4 |  |  |
| rs137926228 | C>A | Intron 1 | 100 | 1 | 0.01 | 0.021 | 0.016 | 0.573 | 4 | lncRNA region | rSNP |
| rs11631342 | A>G | Intron 1 | 100 | 1 | 0.245 | 0.177 | 0.211 | 0.2532 | 4 | lncRNA region | rSNP |
| rs113689788 | G>A | Intron 1 | 100 | 1 | 0.021 | 0.01 | 0.016 | 0.5483 | 4 | lncRNA region | rSNP |
| rs113234775 | T>A | Intron 1 | 100 | 1 | 0.01 | 0.021 | 0.016 | 0.573 | 4 | lncRNA region | rSNP |
| [rs755990193](https://www.ncbi.nlm.nih.gov/projects/SNP/snp_ref.cgi?rs=rs770029099) | G>A | Exon 2 | 98.9 | 1 | 0.011 | 0 | 0.005 | 0.3057 | 4 |  |  |
| rs113174258 | G>A | Exon 2 | 98.9 | 1 | 0.011 | 0 | 0.005 | 0.3057 | 4 |  |  |
| rs7175412 | C>T | Exon 2 | 98.9 | 1 | 0.087 | 0.042 | 0.064 | 0.2041 | 2b |  |  |
| rs11633191 | T>C | Intron 2 | 98.9 | 0.2988 | 0.391 | 0.333 | 0.362 | 0.4083 | 4 |  |  |
| rs7176030 | C>T | Intron 2 | 98.9 | 1 | 0.098 | 0.052 | 0.074 | 0.2324 | 4 |  |  |
| rs2233742 | A>G | Intron 2 | 98.9 | 1 | 0.01 | 0.021 | 0.016 | 0.5858 | 4 |  |  |
| rs2233741 | A>T | Intron 2 | 97.9 | 1 | 0.133 | 0.125 | 0.129 | 0.8655 | 2b |  |  |
| rs2233739 | C>A | Intron 2 | 97.9 | 1 | 0.278 | 0.26 | 0.269 | 0.7896 | 4 |  |  |
| rs866167467* | A>G | Intron 2 | 97.9 | 1 | 0.00 | 0.01 | 0.005 | 0.3316 | 5 |  |  |
| rs867734734* | C>T | Intron 2 | 98.9 | 1 | 0.011 | 0 | 0.005 | 0.3057 | 3a |  |  |
| rs2233737 | A>G | Intron 2 | 97.9 | 1 | 0.01 | 0.021 | 0.016 | 0.5989 | 2b |  |  |
| rs2227300 | G>A | Intron 2 | 98.9 | 0.7278 | 0.10 | 0.104 | 0.101 | 0.8854 | 4 |  |  |
| rs2227298 | C>T | Intron 2 | 97.9 | 1 | 0.00 | 0.011 | 0.005 | 0.3212 | 3a |  |  |
| rs368318275 | ins7>del7 | Intron 2 | 97.9 | 1 | 0.09 | 0.128 | 0.108 | 0.3703 | 3a |  |  |
| rs372553022 | G>T | Intron 2 | 94.7 | 1 | 0.00 | 0.011 | 0.006 | 0.3267 | 4 |  |  |
| rs547080578 | C>T | Intron 2 | 94.7 | 1 | 0.00 | 0.011 | 0.006 | 0.3267 | 4 |  |  |
| rs2233735 | T>C | Intron 2 | 94.7 | 0.5545 | 0.080 | 0.043 | 0.061 | 0.3126 | 4 |  |  |
| rs147602383 | T>C | Intron 2 | 98.9 | 1 | 0.00 | 0.01 | 0.005 | 0.3263 | 3a |  |  |
| rs73412742 | A>C | Intron 2 | 98.9 | 0.4975 | 0.11 | 0.125 | 0.117 | 0.7281 | 4 |  |  |
| rs868184131* | T>C | Intron 2 | 98.9 | 1 | 0.00 | 0.01 | 0.005 | 0.3263 | 5 |  |  |
| rs73412744 | A>G | Intron 2 | 97.9 | 1 | 0.09 | 0.128 | 0.108 | 0.3703 | 5 |  |  |
| rs11631482 | T>A | Intron 2 | 97.9 | 1 | 0.03 | 0.064 | 0.048 | 0.3211 | 5 |  |  |
| rs866994189* | T>A | Intron 2 | 96.8 | 1 | 0.00 | 0.011 | 0.005 | 0.3265 | 5 |  |  |
| rs16940468 | T>G | Intron 2 | 96.8 | 1 | 0.00 | 0.011 | 0.005 | 0.3265 | 5 | circRNA region | rSNP |
| rs41292504 | G>A | Intron 2 | 96.8 | 0.6284 | 0.10 | 0.117 | 0.109 | 0.7108 | 5 | circRNA region | rSNP |
| rs12909325 | G>A | Intron 2 | 96.8 | 1 | 0.00 | 0.021 | 0.011 | 0.1641 | 5 | circRNA region | rSNP |
| rs76588424 | T>C | Intron 2 | 96.8 | 1 | 0.06 | 0.074 | 0.065 | 0.6035 | 5 | circRNA region | rSNP |
| rs59699190 | T>C | Intron 2 | 96.8 | 0.9216 | 0.17 | 0.181 | 0.174 | 0.7997 | 5 | circRNA region | rSNP |
| rs6076 | G>A | Intron 2 | 94.7 | 0.9174 | 0.19 | 0.223 | 0.206 | 0.5356 | 5 | circRNA region | rSNP |
| rs6078 | G>A | Exon 3 | 93.7 | 1 | 0.06 | 0.076 | 0.067 | 0.6332 | 5 |  |  |
| rs776118661 | C>T | Exon 3 | 91.6 | 1 | 0.00 | 0.011 | 0.006 | 0.3215 | 5 |  |  |
| rs373200197 | T>C | Intron 3 | 98.9 | 1 | 0.00 | 0.01 | 0.005 | 0.3263 | 5 |  |  |
| rs865899292* | del3>ins3 | Intron 3 | 96.8 | 0.7966 | 0.09 | 0.109 | 0.098 | 0.6197 | 5 |  |  |
| rs2242061 | T>C | Intron 3 | 96.8 | 1 | 0.15 | 0.196 | 0.174 | 0.4366 | 5 |  |  |
| rs2242062 | A>G | Intron 3 | 96.8 | 1 | 0.11 | 0.152 | 0.13 | 0.3813 | 5 |  |  |
| rs7177852 | C>A | Intron 3 | 95.8 | 1 | 0.022 | 0.022 | 0.022 | 0.9823 | 5 |  |  |
| rs532324326 | G>C | Intron 3 | 95.8 | 1 | 0.011 | 0 | 0.005 | 0.3107 | 5 |  |  |
| rs868007939* | G>A | Intron 3 | 95.8 | 1 | 0.011 | 0 | 0.005 | 0.3107 | 5 |  |  |
| rs690 | T>G | Exon 4 | 95.8 | 0.5817 | 0.44 | 0.5 | 0.473 | 0.4529 | 5 |  |  |
| rs2414589 | C>T | Intron 4 | 96.8 | 0.0492 | 0.09 | 0.098 | 0.092 | 0.799 | 5 |  |  |
| rs2414590 | C>T | Intron 4 | 95.8 | 1 | 0.00 | 0.011 | 0.005 | 0.3319 | 5 |  |  |
| rs369924239 | T>C | Intron 4 | 97.9 | 1 | 0.011 | 0 | 0.005 | 0.3004 | 3a |  |  |
| rs111714545 | T>A | Intron 4 | 98.9 | 1 | 0.022 | 0.01 | 0.016 | 0.5357 | 4 |  |  |
| rs16940472 | C>A | Intron 4 | 97.9 | 0.4257 | 0.43 | 0.458 | 0.446 | 0.7318 | 2b |  |  |
| rs73412750 | C>T | Intron 4 | 96.8 | 1 | 0.01 | 0.032 | 0.022 | 0.3334 | 4 |  |  |
| rs4572327 | T>A | Intron 4 | 96.8 | 1 | 0.21 | 0.266 | 0.239 | 0.3833 | 4 |  |  |
| rs114468383 | A>C | Intron 4 | 97.9 | 1 | 0.011 | 0.01 | 0.011 | 0.9634 | 4 |  |  |
| rs12593954 | T>C | Intron 4 | 96.8 | 1 | 0.05 | 0.094 | 0.071 | 0.2016 | 5 |  |  |
| rs145610869 | T>C | Intron 4 | 96.8 | 1 | 0.00 | 0.021 | 0.011 | 0.1734 | 5 |  |  |
| rs151024681 | G>C | Intron 4 | 96.8 | 1 | 0.023 | 0.021 | 0.022 | 0.9299 | 5 |  |  |
| rs868511461* | T>G | Intron 4 | 95.8 | 1 | 0.00 | 0.01 | 0.005 | 0.3426 | 5 |  |  |
| rs141303471 | G>A | Intron 4 | 96.8 | 1 | 0.023 | 0.021 | 0.022 | 0.9299 | 5 |  |  |
| rs41292508 | A>G | Intron 4 | 96.8 | 1 | 0.102 | 0.062 | 0.082 | 0.3247 | 5 |  |  |
| rs150446650 | G>A | Intron 4 | 95.8 | 1 | 0.023 | 0.021 | 0.022 | 0.9114 | 5 |  |  |
| rs867023819* | G>A | Intron 4 | 95.8 | 1 | 0.023 | 0.01 | 0.016 | 0.497 | 5 |  |  |
| rs11852861 | C>T | Intron 4 | 96.8 | 1 | 0.00 | 0.042 | 0.022 | 0.0529 | 5 |  |  |
| rs78268218 | G>A | Intron 4 | 96.8 | 0.0973 | 0.09 | 0.115 | 0.103 | 0.5981 | 5 |  |  |
| rs144446819 | A>G | Intron 4 | 94.7 | 1 | 0.01 | 0.021 | 0.017 | 0.6135 | 5 |  |  |
| rs56143289 | C>T | Intron 4 | 94.7 | 0.4829 | 0.21 | 0.298 | 0.256 | 0.1736 | 5 |  |  |
| rs573292903 | C>T | Intron 4 | 95.8 | 1 | 0.012 | 0 | 0.005 | 0.2894 | 5 |  |  |
| rs186967434 | G>T | Intron 4 | 95.8 | 1 | 0.023 | 0.021 | 0.022 | 0.9114 | 5 |  |  |
| rs115693448 | G>A | Intron 4 | 95.8 | 1 | 0.01 | 0.062 | 0.038 | 0.0748 | 5 |  |  |
| rs12592139 | G>A | Intron 4 | 95.8 | 1 | 0.02 | 0.094 | 0.06 | 0.0463 | 5 |  |  |
| rs74017973 | G>T | Intron 4 | 97.9 | 1 | 0.03 | 0.094 | 0.065 | 0.0937 | 5 |  |  |
| rs116589263 | G>A | Intron 4 | 97.9 | 1 | 0.022 | 0.01 | 0.016 | 0.523 | 5 |  |  |
| rs12592127 | A>G | Intron 4 | 97.9 | 1 | 0.08 | 0.115 | 0.097 | 0.3962 | 5 |  |  |
| rs140539865 | T>C | Intron 4 | 97.9 | 1 | 0.01 | 0.021 | 0.016 | 0.5989 | 5 |  |  |
| rs17190650 | C>T | Intron 4 | 96.8 | 0.5555 | 0.18 | 0.223 | 0.201 | 0.4402 | 3a |  |  |
| rs2414591 | T>C | Intron 4 | 93.7 | 1 | 0.083 | 0.053 | 0.067 | 0.4233 | 3a |  |  |
| rs11633043 | G>A | Intron 4 | 92.6 | 1 | 0.04 | 0.043 | 0.04 | 0.7924 | 2b |  |  |
| rs4775075 | A>G | Intron 4 | 95.8 | 0.8461 | 0.08 | 0.174 | 0.126 | 0.051 | 4 |  |  |
| rs35200495 | C>G | Intron 4 | 97.9 | 1 | 0.00 | 0.01 | 0.005 | 0.3316 | 4 |  |  |
| rs867530713* | G>A | Intron 4 | 97.9 | 1 | 0.00 | 0.01 | 0.005 | 0.3316 | 4 |  |  |
| rs547226653 | C>T | Intron 4 | 97.9 | 1 | 0.011 | 0.01 | 0.011 | 0.9634 | 3a |  |  |
| rs567104108 | G>A | Intron 4 | 97.9 | 1 | 0.011 | 0.01 | 0.011 | 0.9634 | 3a |  |  |
| rs569556674 | A>C | Intron 4 | 97.9 | 1 | 0.00 | 0.01 | 0.005 | 0.3316 | 2b |  |  |
| rs2414592 | C>T | Intron 4 | 97.9 | 0.8033 | 0.267 | 0.208 | 0.237 | 0.3495 | 3a |  |  |
| rs141216569 | C>T | Intron 4 | 97.9 | 1 | 0.022 | 0.01 | 0.016 | 0.523 | 4 |  |  |
| rs6082 | A>G | Exon 5 | 97.9 | 1 | 0.02 | 0.115 | 0.07 | 0.0136 | 4 |  |  |
| rs6083 | G>A | Exon 5 | 97.9 | 0.8457 | 0.244 | 0.219 | 0.231 | 0.6779 | 4 |  |  |
| rs6084 | C>G | Exon 5 | 97.9 | 1 | 0.200 | 0.188 | 0.194 | 0.8293 | 4 |  |  |
| rs35322472 | G>A | Intron 5 | 96.8 | 1 | 0.034 | 0.031 | 0.033 | 0.9137 | 2b |  |  |
| rs117421299 | C>T | Intron 5 | 96.8 | 1 | 0.011 | 0 | 0.005 | 0.295 | 2b |  |  |
| rs140585049 | C>G | Intron 5 | 98.9 | 1 | 0.033 | 0.031 | 0.032 | 0.9577 | 4 |  |  |
| rs143765093 | G>A | Intron 5 | 96.8 | 1 | 0.011 | 0 | 0.005 | 0.295 | 4 |  |  |
| rs148129322 | G>T | Intron 5 | 98.9 | 1 | 0.022 | 0.01 | 0.016 | 0.5357 | 2a |  |  |
| rs1973023 | C>T | Intron 5 | 96.8 | 0.4833 | 0.19 | 0.26 | 0.228 | 0.2777 | 4 |  |  |
| rs1973024 | C>T | Intron 5 | 98.9 | 0.2003 | 0.23 | 0.271 | 0.25 | 0.5004 | 4 |  |  |
| rs2242063 | G>A | Intron 5 | 95.8 | 1 | 0.078 | 0.065 | 0.071 | 0.7422 | 4 |  |  |
| rs560800241 | G>A | Intron 5 | 95.8 | 1 | 0.011 | 0 | 0.005 | 0.3107 | 4 |  |  |
| rs7181352 | C>G | Intron 5 | 95.8 | 0.7343 | 0.21 | 0.217 | 0.214 | 0.9178 | 4 |  |  |
| rs116502523 | A>G | Intron 5 | 95.8 | 1 | 0.011 | 0.011 | 0.011 | 0.9875 | 4 |  |  |
| rs10459627 | T>C | Intron 5 | 95.8 | 1 | 0.02 | 0.043 | 0.033 | 0.422 | 4 |  |  |
| rs2242064 | T>G | Intron 5 | 91.6 | 0.2639 | 0.29 | 0.311 | 0.299 | 0.7146 | 4 |  |  |
| rs2242065 | C>T | Intron 5 | 91.6 | 1 | 0.02 | 0.089 | 0.057 | 0.0653 | 2a |  |  |
| rs2242066 | G>A | Intron 5 | 91.6 | 1 | 0.238 | 0.178 | 0.207 | 0.3263 | 3a |  |  |
| rs34519838 | C>T | Intron 5 | 92.6 | 1 | 0.023 | 0.011 | 0.017 | 0.5338 | 4 |  |  |
| rs868284719* | G>A | Intron 5 | 92.6 | 1 | 0.012 | 0 | 0.006 | 0.3049 | 2b |  |  |
| rs114127971 | G>A | Intron 5 | 92.6 | 1 | 0.00 | 0.011 | 0.006 | 0.3269 | 4 |  |  |
| rs534777002 | G>A | Intron 5 | 92.6 | 1 | 0.012 | 0 | 0.006 | 0.3049 | 4 |  |  |
| rs114971017 | G>A | Intron 5 | 92.6 | 1 | 0.05 | 0.056 | 0.051 | 0.7854 | 4 |  |  |
| rs112147431 | C>T | Intron 5 | 40 | 1 | 0.00 | 0.025 | 0.013 | 0.3396 | 2b |  |  |
| rs372284932 | A>G | Intron 5 | 37.9 | 1 | 0.00 | 0.053 | 0.028 | 0.1749 | 4 |  |  |
| rs143731122 | ins5>del5 | Intron 5 | 92.6 | 0.0259 | 0.35 | 0.426 | 0.392 | 0.3299 | 4 |  |  |
| rs7165654 | G>A | Intron 5 | 96.8 | 0.0218 | 0.38 | 0.479 | 0.429 | 0.1667 | 4 |  |  |
| rs144554638 | C>T | Intron 5 | 97.9 | 1 | 0.033 | 0.031 | 0.032 | 0.9359 | 4 |  |  |
| rs12913969 | G>A | Intron 5 | 97.9 | 1 | 0.06 | 0.062 | 0.059 | 0.841 | 4 |  |  |
| rs74018854 | C>T | Intron 5 | 97.9 | 1 | 0.00 | 0.042 | 0.022 | 0.0503 | 5 |  |  |
| rs1007543 | C>A | Intron 5 | 97.9 | 1 | 0.222 | 0.219 | 0.22 | 0.9545 | 5 |  |  |
| rs74018855 | T>C | Intron 5 | 97.9 | 1 | 0.00 | 0.042 | 0.022 | 0.0503 | 4 |  |  |
| rs146299102 | C>T | Exon 6 | 97.9 | 1 | 0.022 | 0.021 | 0.022 | 0.948 | 4 |  |  |
| rs145811475 | G>A | Exon 6 | 100 | 1 | 0.011 | 0 | 0.005 | 0.3109 | 4 |  |  |
| rs1973025 | C>G | Intron 6 | 98.9 | 0.9563 | 0.174 | 0.167 | 0.17 | 0.8949 | 5 |  |  |
| rs1973026 | T>A | Intron 6 | 98.9 | 0.9563 | 0.174 | 0.167 | 0.17 | 0.8949 | 5 |  |  |
| rs1973027 | C>T | Intron 6 | 98.9 | 0.9563 | 0.174 | 0.167 | 0.17 | 0.8949 | 5 |  |  |
| rs180818510 | T>G | Intron 6 | 98.9 | 1 | 0.01 | 0.021 | 0.016 | 0.5858 | 5 |  |  |
| rs116534057 | C>T | Intron 6 | 97.9 | 1 | 0.01 | 0.021 | 0.016 | 0.5989 | 4 |  |  |
| rs533377312 | A>G | Intron 6 | 98.9 | 1 | 0.011 | 0 | 0.005 | 0.3057 | 4 |  |  |
| rs7166788 | G>C | Intron 6 | 97.9 | 0.2966 | 0.315 | 0.298 | 0.306 | 0.7975 | 4 |  |  |
| rs4775077 | G>A | Intron 6 | 96.8 | 0.8223 | 0.182 | 0.177 | 0.179 | 0.9334 | 5 |  |  |
| rs544754564 | C>A | Intron 6 | 96.8 | 1 | 0.00 | 0.01 | 0.005 | 0.337 | 5 |  |  |
| rs569105133 | A>T | Intron 6 | 100 | 1 | 0.00 | 0.01 | 0.005 | 0.3211 | 5 |  |  |
| rs531491245 | C>G | Intron 6 | 96.8 | 1 | 0.00 | 0.01 | 0.005 | 0.337 | 5 |  |  |
| rs551190960 | A>C | Intron 6 | 100 | 1 | 0.00 | 0.01 | 0.005 | 0.3211 | 5 |  |  |
| rs867359212* | C>T | Intron 6 | 97.9 | 0.0108 | 0.00 | 0.021 | 0.011 | 0.1686 | 5 |  |  |
| rs79086243 | T>C | Intron 6 | 98.9 | 1 | 0.03 | 0.042 | 0.037 | 0.743 | 5 |  |  |
| rs867552391* | G>A | Intron 6 | 98.9 | 1 | 0.011 | 0 | 0.005 | 0.3057 | 5 |  |  |
| rs150007052 | T>C | Intron 6 | 98.9 | 1 | 0.01 | 0.021 | 0.016 | 0.5858 | 5 |  |  |
| rs1007542 | G>A | Intron 6 | 98.9 | 1 | 0.174 | 0.156 | 0.165 | 0.7442 | 5 |  |  |
| rs11632627 | A>G | Intron 6 | 98.9 | 0.0348 | 0.500 | 0.417 | 0.457 | 0.2516 | 5 |  |  |
| rs183706349 | T>G | Intron 6 | 97.9 | 6.12E-10 | 0.00 | 0.125 | 0.065 | 5.00E-04 | 5 |  |  |
| rs56171273 | G>A | Intron 6 | 68.4 | 1 | 0.02 | 0.016 | 0.015 | 0.9825 | 6 |  |  |
| rs11632796 | G>C | Intron 6 | 67.4 | 0.3399 | 0.219 | 0.203 | 0.211 | 0.8285 | 6 |  |  |
| rs542752846 | A>G | Intron 6 | 66.3 | 1 | 0.00 | 0.016 | 0.008 | 0.3231 | 6 |  |  |
| rs56122478 | del8>ins8 | Intron 6 | 96.8 | 0.6436 | 0.156 | 0.089 | 0.122 | 0.1721 | 5 |  |  |
| rs67727760 | C>T | Intron 6 | 94.7 | 0.2338 | 0.10 | 0.128 | 0.114 | 0.5553 | 5 |  |  |
| rs78861621 | C>T | Intron 6 | 91.6 | 1 | 0.13 | 0.159 | 0.144 | 0.5577 | 5 |  |  |
| rs942481496 | C>T | Intron 6 | 81.1 | 1 | 0.013 | 0 | 0.006 | 0.3094 | 5 |  |  |
| rs866164447* | A>T | Intron 6 | 96.8 | 1 | 0.011 | 0 | 0.005 | 0.316 | 4 |  |  |
| rs74576231 | T>C | Intron 6 | 96.8 | 1 | 0.033 | 0.022 | 0.027 | 0.6502 | 2b |  |  |
| rs866659155* | A>C | Intron 6 | 97.9 | 1 | 0.011 | 0 | 0.005 | 0.3212 | 4 |  |  |
| rs142884409 | C>G | Intron 6 | 97.9 | 1 | 0.02 | 0.032 | 0.027 | 0.6679 | 4 |  |  |
| rs532159628 | T>C | Intron 6 | 100 | 1 | 0.011 | 0 | 0.005 | 0.3109 | 4 |  |  |
| rs871804 | C>T | Intron 6 | 100 | 1 | 0.074 | 0.073 | 0.074 | 0.9674 | 4 |  |  |
| rs151058229 | C>A | Intron 6 | 100 | 1 | 0.021 | 0 | 0.011 | 0.1508 | 4 |  |  |
| rs139739105 | G>C | Intron 6 | 98.9 | 1 | 0.022 | 0.01 | 0.016 | 0.5357 | 4 |  |  |
| rs1869146 | G>A | Intron 6 | 98.9 | 0.2254 | 0.14 | 0.146 | 0.144 | 0.9295 | 2b |  |  |
| rs867531593* | G>A | Intron 6 | 98.9 | 1 | 0.00 | 0.01 | 0.005 | 0.3263 | 2c |  |  |
| rs866494468* | C>T | Intron 6 | 98.9 | 1 | 0.011 | 0 | 0.005 | 0.3057 | 4 |  |  |
| rs866589210* | G>T | Intron 6 | 96.8 | 1 | 0.011 | 0 | 0.005 | 0.316 | 4 |  |  |
| rs41292510 | T>A | Intron 6 | 97.9 | 1 | 0.067 | 0.042 | 0.054 | 0.45 | 4 |  |  |
| rs114189657 | G>A | Intron 6 | 97.9 | 1 | 0.044 | 0.042 | 0.043 | 0.9257 | 5 |  |  |
| rs147112431 | C>T | Intron 6 | 97.9 | 1 | 0.00 | 0.021 | 0.011 | 0.1686 | 5 |  |  |
| rs148818173 | G>A | Intron 6 | 93.7 | 1 | 0.023 | 0.011 | 0.017 | 0.5472 | 4 |  |  |
| rs12908645 | T>C | Intron 6 | 93.7 | 1 | 0.07 | 0.078 | 0.073 | 0.8057 | 3a |  |  |
| rs79791400 | A>G | Intron 6 | 93.7 | 1 | 0.023 | 0.011 | 0.017 | 0.5472 | 3a |  |  |
| rs45500398 | T>A | Intron 6 | 93.7 | 0.2305 | 0.00 | 0.078 | 0.039 | 0.0076 | 5 |  |  |
| rs57437914 | A>G | Intron 6 | 98.9 | 0.3636 | 0.02 | 0.073 | 0.048 | 0.1004 | 5 |  |  |
| rs16940493 | C>T | Intron 6 | 98.9 | 1 | 0.04 | 0.073 | 0.059 | 0.39 | 5 |  |  |
| rs115916514 | G>A | Intron 6 | 97.9 | 1 | 0.03 | 0.052 | 0.043 | 0.5288 | 5 |  |  |
| rs12595265 | G>C | Intron 6 | 94.7 | 1 | 0.02 | 0.062 | 0.044 | 0.2089 | 5 |  |  |
| rs11071389 | G>T | Intron 6 | 95.8 | 0.1244 | 0.34 | 0.385 | 0.363 | 0.4994 | 5 |  |  |
| rs557314024 | G>T | Intron 6 | 97.9 | 1 | 0.00 | 0.01 | 0.005 | 0.3316 | 5 |  |  |
| rs28524122 | G>A | Intron 6 | 97.9 | 1 | 0.067 | 0.062 | 0.065 | 0.908 | 5 |  |  |
| rs139270417 | C>T | Intron 6 | 97.9 | 1 | 0.044 | 0.042 | 0.043 | 0.9257 | 5 |  |  |
| rs868768501* | G>A | Intron 6 | 98.9 | 1 | 0.011 | 0 | 0.005 | 0.3057 | 5 |  |  |
| rs7178362 | T>C | Intron 6 | 97.9 | 1 | 0.12 | 0.135 | 0.129 | 0.7885 | 5 |  |  |
| rs17190678 | C>G | Intron 6 | 98.9 | 0.7278 | 0.109 | 0.094 | 0.101 | 0.734 | 5 |  |  |
| rs149566194 | C>T | Intron 6 | 97.9 | 1 | 0.022 | 0 | 0.011 | 0.142 | 5 |  |  |
| rs16940495 | C>G | Intron 6 | 98.9 | 1 | 0.043 | 0.031 | 0.037 | 0.658 | 5 |  |  |
| rs147941779 | G>A | Intron 6 | 93.7 | 1 | 0.024 | 0 | 0.011 | 0.1238 | 3a |  |  |
| rs140083043 | T>C | Intron 6 | 93.7 | 1 | 0.049 | 0.042 | 0.045 | 0.8194 | 4 |  |  |
| rs41294813 | T>C | Intron 6 | 96.8 | 0.7102 | 0.114 | 0.094 | 0.103 | 0.6579 | 3a |  |  |
| rs116529755 | C>A | Intron 6 | 97.9 | 0.2904 | 0.044 | 0.042 | 0.043 | 0.9257 | 2b |  |  |
| rs78312967 | G>A | Intron 6 | 97.9 | 0.3944 | 0.16 | 0.188 | 0.172 | 0.564 | 4 |  |  |
| rs56010348 | T>G | Intron 6 | 97.9 | 0.8053 | 0.111 | 0.083 | 0.097 | 0.5219 | 2c |  |  |
| rs192359789 | G>A | Intron 6 | 97.9 | 1 | 0.011 | 0 | 0.005 | 0.3004 | 4 |  |  |
| rs544724399 | C>A | Intron 6 | 97.9 | 1 | 0.011 | 0.01 | 0.011 | 0.9634 | 4 |  |  |
| rs187956881 | G>A | Intron 6 | 96.8 | 1 | 0.011 | 0 | 0.005 | 0.3055 | 4 |  |  |
| rs74016903 | C>T | Intron 6 | 96.8 | 0.1084 | 0.00 | 0.053 | 0.027 | 0.0265 | 4 |  |  |
| rs41294815 | A>G | Intron 6 | 96.8 | 0.3197 | 0.06 | 0.128 | 0.092 | 0.0913 | 4 |  |  |
| rs865875454* | C>T | Intron 6 | 97.9 | 1 | 0.00 | 0.011 | 0.005 | 0.3212 | 3a |  |  |
| rs7166817 | G>A | Intron 6 | 97.9 | 1 | 0.043 | 0.032 | 0.038 | 0.6786 | 3a |  |  |
| rs531486487 | T>C | Intron 6 | 97.9 | 1 | 0.011 | 0 | 0.005 | 0.3108 | 2b |  |  |
| rs547133456 | C>T | Intron 6 | 98.9 | 1 | 0.00 | 0.01 | 0.005 | 0.3263 | 2b |  |  |
| rs6494018 | G>T | Intron 6 | 98.9 | 0.2323 | 0.49 | 0.49 | 0.489 | 0.995 | 2b |  |  |
| rs75649447 | G>A | Intron 6 | 97.9 | 1 | 0.011 | 0 | 0.005 | 0.3004 | 5 |  |  |
| rs189007731 | T>C | Intron 6 | 97.9 | 1 | 0.022 | 0.021 | 0.022 | 0.948 | 4 |  |  |
| rs35631005 | C>T | Intron 6 | 97.9 | 1 | 0.011 | 0 | 0.005 | 0.3004 | 4 |  |  |
| rs7173774 | C>T | Intron 6 | 91.6 | 1 | 0.116 | 0.08 | 0.098 | 0.4145 | 5 |  |  |
| rs529784089 | G>A | Intron 6 | 91.6 | 1 | 0.012 | 0 | 0.006 | 0.3104 | 5 |  |  |
| rs35892254 | A>G | Intron 6 | 91.6 | 1 | 0.04 | 0.08 | 0.057 | 0.2057 | 5 |  |  |
| rs72743041 | C>T | Intron 6 | 95.8 | 3.09E-14 | 0.216 | 0.149 | 0.181 | 0.2412 | NA |  |  |
| rs866795800* | C>G | Intron 6 | 77.9 | 1 | 0.014 | 0 | 0.007 | 0.2895 | 6 |  |  |
| rs538611715 | C>A | Intron 6 | 87.4 | 1 | 0.026 | 0 | 0.012 | 0.1307 | 7 |  |  |
| rs397715435 | del1>ins1 | Intron 6 | 95.8 | 1 | 0.10 | 0.106 | 0.104 | 0.9278 | 7 |  |  |
| rs534915892 | C>G | Intron 6 | 95.8 | 1 | 0.01 | 0.021 | 0.016 | 0.5997 | 5 |  |  |
| rs2414593 | C>T | Intron 6 | 97.9 | 0.652 | 0.18 | 0.198 | 0.188 | 0.7255 | 5 |  |  |
| rs2414594 | G>A | Intron 6 | 96.8 | 0.5486 | 0.19 | 0.198 | 0.196 | 0.9355 | 5 |  |  |
| rs61207362* | ins30>del30 | Intron 6 | 97.9 | 0.878 | 0.233 | 0.177 | 0.204 | 0.3417 | 5 |  |  |
| rs2414595* | C>A | Intron 6 | 97.9 | 0.0789 | 0.17 | 0.177 | 0.172 | 0.8508 | 5 |  |  |
| rs2414596 | G>C | Intron 6 | 97.9 | 0.5653 | 0.19 | 0.198 | 0.194 | 0.8762 | 5 |  |  |
| rs2414597 | C>A | Intron 6 | 67.4 | 0.0451 | 0.078 | 0.062 | 0.07 | 0.7296 | 4 |  |  |
| rs748876111 | C>T | Intron 6 | 96.8 | 1 | 0.01 | 0.021 | 0.016 | 0.6124 | 4 |  |  |
| rs112246374 | A>G | Intron 6 | 95.8 | 0.0661 | 0.035 | 0.01 | 0.022 | 0.261 | 4 |  |  |
| rs2414598 | A>G | Intron 6 | 95.8 | 0.9039 | 0.16 | 0.188 | 0.176 | 0.662 | 4 |  |  |
| rs868712542* | A>T | Intron 6 | 94.7 | 1 | 0.00 | 0.011 | 0.006 | 0.3375 | 3a |  |  |
| rs865809947* | ins2>del2 | Intron 6 | 95.8 | 1 | 0.01 | 0.021 | 0.016 | 0.6263 | 4 |  |  |
| rs140120301 | C>T | Intron 6 | 92.6 | 1 | 0.012 | 0 | 0.006 | 0.2939 | 4 |  |  |
| rs78884173 | C>G | Intron 6 | 92.6 | 0.0684 | 0.036 | 0.011 | 0.023 | 0.2693 | 4 |  |  |
| rs2414599 | T>C | Intron 6 | 92.6 | 1 | 0.16 | 0.174 | 0.165 | 0.7323 | 4 |  |  |
| rs2414600 | A>C | Intron 6 | 91.6 | 1 | 0.14 | 0.167 | 0.155 | 0.6647 | 3a |  |  |
| rs2414601 | T>C | Intron 6 | 91.6 | 1 | 0.16 | 0.167 | 0.161 | 0.8309 | 3a |  |  |
| rs144323552 | T>A | Intron 6 | 91.6 | 1 | 0.036 | 0.033 | 0.034 | 0.9315 | 4 |  |  |
| rs28455962 | T>A | Intron 6 | 91.6 | 0.9623 | 0.17 | 0.178 | 0.172 | 0.8463 | 5 |  |  |
| rs4444272 | C>A | Intron 6 | 93.7 | 1 | 0.15 | 0.185 | 0.169 | 0.5493 | 3a |  |  |
| rs4528512 | T>A | Intron 6 | 93.7 | 0.069 | 0.384 | 0.337 | 0.36 | 0.5159 | 3a |  |  |
| rs187151902 | G>A | Intron 6 | 95.8 | 1 | 0.00 | 0.021 | 0.011 | 0.1688 | 4 |  |  |
| rs867924619* | A>T | Intron 6 | 95.8 | 1 | 0.011 | 0 | 0.005 | 0.3 | 4 |  |  |
| rs11858020 | A>T | Intron 6 | 94.7 | 1 | 0.151 | 0.096 | 0.122 | 0.2569 | 4 |  |  |
| rs7179747 | C>A | Intron 6 | 95.8 | 1 | 0.15 | 0.181 | 0.165 | 0.5473 | 2b |  |  |
| rs7179940 | G>C | Intron 6 | 95.8 | 1 | 0.15 | 0.181 | 0.165 | 0.5473 | 4 |  |  |
| rs142451556 | T>C | Intron 6 | 95.8 | 0.1096 | 0.034 | 0.021 | 0.027 | 0.5971 | 2b |  |  |
| rs7181367 | C>T | Intron 6 | 95.8 | 1 | 0.15 | 0.181 | 0.165 | 0.5473 | 2b |  |  |
| rs7179938 | C>A | Intron 6 | 95.8 | 1 | 0.15 | 0.181 | 0.165 | 0.5473 | 3a |  |  |
| rs11639204 | A>G | Intron 6 | 95.8 | 1 | 0.15 | 0.181 | 0.165 | 0.5473 | 2a |  |  |
| rs7180130 | G>A | Intron 6 | 98.9 | 0.9563 | 0.15 | 0.188 | 0.17 | 0.5194 | 4 |  |  |
| rs7181592 | C>T | Intron 6 | 98.9 | 0.7614 | 0.16 | 0.198 | 0.181 | 0.5346 | 2b |  |  |
| rs7180795 | A>G | Intron 6 | 98.9 | 0.7614 | 0.16 | 0.198 | 0.181 | 0.5346 | 5 |  |  |
| rs7181945 | G>T | Intron 6 | 98.9 | 0.7614 | 0.16 | 0.198 | 0.181 | 0.5346 | 5 |  |  |
| rs71425810 | del20>ins20 | Intron 6 | 98.9 | 0.7614 | 0.16 | 0.198 | 0.181 | 0.5346 | 6 |  |  |
| rs33931419 | G>C | Intron 6 | 67.4 | 0.9033 | 0.470 | 0.371 | 0.422 | 0.2583 | 7 |  |  |
| rs3829463 | C>A | Intron 6 | 67.4 | 4.00E-04 | 0.03 | 0.032 | 0.031 | 0.9493 | 7 |  |  |
| rs140777851 | C>G | Intron 6 | 67.4 | 1 | 0.045 | 0 | 0.023 | 0.0894 | 7 |  |  |
| rs3829462 | A>C | Exon 7 | 68.4 | 4.00E-04 | 0.03 | 0.032 | 0.031 | 0.9252 | 6 | circRNA region | rSNP |
| rs3829461 | G>A | Exon 7 | 68.4 | 4.00E-04 | 0.03 | 0.032 | 0.031 | 0.9252 | 3a | circRNA region | rSNP |
| rs868805990* | del2>ins2 | Intron 7 | 95.8 | 0.2484 | 0.13 | 0.152 | 0.143 | 0.7165 | 2b |  |  |
| rs368337583 | C>G | Intron 7 | 96.8 | 0.7966 | 0.09 | 0.109 | 0.098 | 0.6197 | 2b | circRNA region | rSNP |
| rs72062747 | del2>ins2 | Intron 7 | 97.9 | 0.7439 | 0.17 | 0.191 | 0.183 | 0.7565 | 2b |  |  |
| rs55913623 | T>C | Intron 7 | 32.6 | 0.5403 | 0.36 | 0.471 | 0.419 | 0.3677 | 4 | circRNA region | rSNP |
| [rs533300601](https://www.ncbi.nlm.nih.gov/projects/SNP/snp_ref.cgi?rs=rs533300601) | G>A | Intron 7 | 97.9 | 0.0324 | 0.033 | 0 | 0.016 | 0.0776 | 3a | circRNA region | rSNP |
| rs546538665 | A>T | Intron 7 | 97.9 | 0.0324 | 0.033 | 0 | 0.016 | 0.0776 | 3a |  |  |
| rs4774305 | G>C | Intron 7 | 98.9 | 0.7614 | 0.16 | 0.198 | 0.181 | 0.5346 | 4 | circRNA region | rSNP |
| rs568646677 | G>T | Intron 7 | 98.9 | 1 | 0.011 | 0 | 0.005 | 0.3057 | 4 | circRNA region | rSNP |
| rs1869129 | T>C | Intron 7 | 98.9 | 0.3056 | 0.24 | 0.24 | 0.239 | 0.9942 | 3a | circRNA region | rSNP |
| rs115408618 | G>C | Intron 7 | 96.8 | 1 | 0.023 | 0 | 0.011 | 0.1375 | 3a | circRNA region | rSNP |
| rs1869130 | C>T | Intron 7 | 96.8 | 0.7263 | 0.17 | 0.198 | 0.185 | 0.6316 | 4 | circRNA region | rSNP |
| rs12438032 | G>A | Intron 7 | 97.9 | 1 | 0.16 | 0.156 | 0.156 | 0.9896 | 5 | circRNA region | rSNP |
| rs34964641 | T>G | Intron 7 | 97.9 | 0.7439 | 0.17 | 0.198 | 0.183 | 0.5816 | 5 | circRNA region | rSNP |
| rs1869131 | A>T | Intron 7 | 94.7 | 0.9878 | 0.17 | 0.178 | 0.172 | 0.8435 | 5 | circRNA region | rSNP |
| rs4775079 | C>T | Intron 7 | 95.8 | 0.9039 | 0.17 | 0.185 | 0.176 | 0.7482 | 5 | circRNA region | rSNP |
| rs1869132 | T>G | Intron 7 | 95.8 | 0.0854 | 0.411 | 0.359 | 0.385 | 0.4674 | 5 | circRNA region | rSNP |
| rs8026372 | A>G | Intron 7 | 88.4 | 1 | 0.151 | 0.146 | 0.149 | 0.9301 | 5 | circRNA region | rSNP |
| rs1839928 | A>G | Intron 7 | 98.9 | 0.7614 | 0.16 | 0.202 | 0.181 | 0.4485 | 5 | circRNA region | rSNP |
| rs1839927 | A>G | Intron 7 | 98.9 | 0.7614 | 0.16 | 0.202 | 0.181 | 0.4485 | 5 | circRNA region | rSNP |
| rs8027708 | A>T | Intron 7 | 98.9 | 0.0958 | 0.362 | 0.362 | 0.362 | 1 | 5 | circRNA region | rSNP |
| rs36017602 | G>A | Intron 7 | 98.9 | 1 | 0.117 | 0.117 | 0.117 | 1 | 4 | circRNA region | rSNP |
| rs8030893 | G>C | Intron 7 | 98.9 | 0.0958 | 0.362 | 0.362 | 0.362 | 1 | 4 | circRNA region | rSNP |
| rs8030903 | T>C | Intron 7 | 98.9 | 0.7614 | 0.16 | 0.202 | 0.181 | 0.4485 | 4 | circRNA region | rSNP |
| rs10851636 | C>T | Intron 7 | 98.9 | 0.7614 | 0.16 | 0.202 | 0.181 | 0.4485 | 4 | circRNA region | rSNP |
| rs10851637 | C>G | Intron 7 | 98.9 | 0.0958 | 0.362 | 0.362 | 0.362 | 1 | 4 | circRNA region | rSNP |
| rs7170227 | G>A | Intron 7 | 100 | 0.7787 | 0.16 | 0.198 | 0.179 | 0.4906 | 3a | circRNA region | rSNP |
| rs143889538 | G>A | Intron 7 | 98.9 | 1 | 0.021 | 0 | 0.011 | 0.1551 | 2b | circRNA region | rSNP |
| rs190375050 | C>T | Intron 7 | 98.9 | 1 | 0.00 | 0.021 | 0.011 | 0.164 | 5 | circRNA region | rSNP |
| rs72038414 | C>G | Intron 7 | 98.9 | 1 | 0.043 | 0.031 | 0.037 | 0.658 | NA |  |  |
| rs866156204* | C>T | Intron 7 | 98.9 | 1 | 0.011 | 0.01 | 0.011 | 0.9759 | 5 |  |  |
| rs17301857 | A>G | Intron 7 | 98.9 | 1 | 0.120 | 0.115 | 0.117 | 0.9154 | 5 | circRNA region | rSNP |
| rs35412158 | G>A | Intron 7 | 98.9 | 1 | 0.04 | 0.073 | 0.059 | 0.39 | 5 | circRNA region | rSNP |
| rs7171818 | T>A | Intron 7 | 97.9 | 9.47E-06 | 0.09 | 0.125 | 0.108 | 0.4269 | 5 |  |  |
|  | G>A |  | 97.9 | 0.6334 | 0.078 | 0.073 | 0.075 | 0.5903 | 5 | circRNA region | rSNP |
|  | G>T |  | 97.9 | 0.0014 | 0.10 | 0.125 | 0.113 | 0.9001 | 5 |  |  |
| rs17301864 | C>T | Intron 7 | 93.7 | 1 | 0.128 | 0.12 | 0.124 | 0.8658 | 5 | circRNA region | rSNP |
| rs11071390 | A>G | Intron 7 | 98.9 | 0.9563 | 0.16 | 0.181 | 0.17 | 0.6979 | 5 | circRNA region | rSNP |
| rs181084356 | C>G | Intron 7 | 98.9 | 1 | 0.00 | 0.021 | 0.011 | 0.1551 | 5 | circRNA region | rSNP |
| rs145482805 | ins1>del1 | Intron 7 | 98.9 | 1 | 0.04 | 0.096 | 0.069 | 0.1506 | 5 | circRNA region | rSNP |
| rs7176457 | G>A | Intron 7 | 98.9 | 0.7153 | 0.117 | 0.117 | 0.117 | 1 | 5 | circRNA region | rSNP |
| rs75983069 | A>G | Exon 8 | 97.9 | 1 | 0.02 | 0.032 | 0.027 | 0.6679 | 5 | circRNA region | rSNP |
| rs3751542 | C>T | Intron 8 | 95.8 | 0.3295 | 0.46 | 0.457 | 0.456 | 0.9896 | 5 |  |  |
| rs6494019 | T>C | Intron 8 | 94.7 | 0.5728 | 0.11 | 0.117 | 0.111 | 0.7919 | 5 |  |  |
| rs6494020 | C>T | Intron 8 | 95.8 | 0.654 | 0.11 | 0.117 | 0.115 | 0.9431 | 5 |  |  |
| rs6494021 | T>G | Intron 8 | 95.8 | 0.5632 | 0.10 | 0.117 | 0.11 | 0.7505 | 5 |  |  |
| rs6494022 | A>G | Intron 8 | 95.8 | 0.5632 | 0.10 | 0.117 | 0.11 | 0.7505 | 5 |  |  |
| rs6494023 | T>A | Intron 8 | 95.8 | 0.3531 | 0.11 | 0.149 | 0.132 | 0.4818 | 5 |  |  |
| rs8038420 | A>G | Intron 8 | 94.7 | 0.6647 | 0.11 | 0.12 | 0.117 | 0.9014 | 5 |  |  |
| rs75472403 | A>G | Intron 8 | 55.8 | 1 | 0.04 | 0.089 | 0.066 | 0.3077 | 5 |  |  |
| rs12440615 | A>C | Intron 8 | 54.7 | 0.0583 | 0.042 | 0.018 | 0.029 | 0.4696 | 5 |  |  |
| rs4288938 | A>G | Intron 8 | 54.7 | 6.00E-04 | 0.042 | 0.036 | 0.038 | 0.875 | 5 |  |  |
| rs4420471 | T>A | Intron 8 | 54.7 | 6.00E-04 | 0.042 | 0.036 | 0.038 | 0.875 | 5 |  |  |
| rs150176124 | C>T | Intron 8 | 55.8 | 0.1137 | 0.060 | 0.018 | 0.038 | 0.2557 | 5 |  |  |
| rs67897154 | G>A | Intron 8 | 55.8 | 0.6318 | 0.14 | 0.143 | 0.142 | 0.9664 | 5 |  |  |
| rs66467237 | ins1>del1 | Intron 8 | 65.3 | 0.3994 | 0.12 | 0.121 | 0.121 | 0.9929 | 5 |  |  |
| rs141409911 | A>G | Intron 8 | 66.3 | 1 | 0.069 | 0 | 0.032 | 0.0278 | 5 |  |  |
| rs2899633 | C>A | Intron 8 | 65.3 | 0.3994 | 0.12 | 0.121 | 0.121 | 0.9929 | 7 |  |  |
| rs138285708 | C>T | Intron 8 | 66.3 | 1 | 0.086 | 0 | 0.04 | 0.0135 | 6 |  |  |
| rs184104751 | G>A | Intron 8 | 97.9 | 1 | 0.022 | 0.01 | 0.016 | 0.523 | 6 |  |  |
| rs2414602 | A>G | Intron 8 | 94.7 | 0.5728 | 0.114 | 0.109 | 0.111 | 0.916 | 7 |  |  |
| rs185850072 | G>A | Intron 8 | 97.9 | 1 | 0.00 | 0.021 | 0.011 | 0.1686 | 7 |  |  |
| rs2414603 | G>A | Intron 8 | 97.9 | 0.8219 | 0.12 | 0.125 | 0.124 | 0.9541 | 5 |  |  |
| rs186982828 | A>C | Intron 8 | 97.9 | 1 | 0.044 | 0.031 | 0.038 | 0.6365 | 5 |  |  |
| rs143278180 | G>A | Intron 8 | 97.9 | 1 | 0.033 | 0.01 | 0.022 | 0.2816 | 5 |  |  |
| rs148359717 | C>T | Intron 8 | 97.9 | 1 | 0.011 | 0.01 | 0.011 | 0.9634 | 5 |  |  |
| rs115279026 | G>A | Intron 8 | 97.9 | 1 | 0.01 | 0.021 | 0.016 | 0.5989 | 5 |  |  |
| rs114323767 | C>T | Intron 8 | 97.9 | 1 | 0.044 | 0.031 | 0.038 | 0.6365 | 5 |  |  |
| rs7163112 | G>T | Intron 8 | 97.9 | 0.8219 | 0.12 | 0.125 | 0.124 | 0.9541 | 4 |  |  |
| rs4775080 | C>G | Intron 8 | 97.9 | 0.8219 | 0.12 | 0.125 | 0.124 | 0.9541 | 3a |  |  |
| rs4775081 | G>A | Intron 8 | 97.9 | 0.8219 | 0.12 | 0.125 | 0.124 | 0.9541 | 4 |  |  |
| rs115464904 | C>T | Intron 8 | 97.9 | 1 | 0.056 | 0.052 | 0.054 | 0.9164 | 4 |  |  |
| rs17269397 | A>G | Intron 8 | 88.4 | 0.4794 | 0.125 | 0.125 | 0.125 | 1 | 4 |  |  |
| rs575521393 | G>A | Intron 8 | 89.5 | 1 | 0.012 | 0 | 0.006 | 0.2988 | 4 |  |  |
| rs114990659 | T>C | Intron 8 | 89.5 | 1 | 0.012 | 0 | 0.006 | 0.2988 | 4 |  |  |
| rs867729837* | C>A | Intron 8 | 89.5 | 1 | 0.00 | 0.011 | 0.006 | 0.333 | 4 |  |  |
| rs41294817 | A>G | Intron 8 | 88.4 | 1 | 0.025 | 0.011 | 0.018 | 0.5051 | 5 |  |  |
| rs3829460 | A>T | Intron 8 | 88.4 | 0.5681 | 0.28 | 0.284 | 0.28 | 0.8957 | 5 |  |  |
| rs7169280 | C>T | Intron 8 | 95.8 | 0.7488 | 0.12 | 0.125 | 0.121 | 0.857 | 5 |  |  |
| rs7168293 | A>G | Intron 8 | 95.8 | 0.654 | 0.116 | 0.115 | 0.115 | 0.9715 | 5 |  |  |
| rs10152558 | T>C | Intron 8 | 89.5 | 0.4887 | 0.12 | 0.125 | 0.124 | 0.9519 | 3a |  |  |
| rs139092954 | C>A | Intron 8 | 90.5 | 0.0351 | 0.038 | 0 | 0.017 | 0.061 | 4 |  |  |
| rs575521393 | C>T | Intron 8 | 91.6 | 1 | 0.012 | 0 | 0.006 | 0.2881 | 4 |  |  |
| rs28602186 | G>A | Intron 8 | 91.6 | 1 | 0.098 | 0.054 | 0.075 | 0.2792 | 4 |  |  |
| rs144940322 | T>G | Intron 8 | 91.6 | 1 | 0.024 | 0.011 | 0.017 | 0.494 | 3a |  |  |
| rs16940537 | T>C | Intron 8 | 91.6 | 1 | 0.049 | 0.043 | 0.046 | 0.8676 | 7 |  |  |
| rs115278822 | C>A | Intron 8 | 100 | 1 | 0.021 | 0.01 | 0.016 | 0.5483 | 5 |  |  |
| rs7175421 | C>T | Intron 8 | 95.8 | 0.3068 | 0.456 | 0.435 | 0.445 | 0.778 | 5 |  |  |
| rs11637823 | T>C | Intron 8 | 95.8 | 0.8461 | 0.12 | 0.13 | 0.126 | 0.8676 | 5 |  |  |
| rs114687875 | C>T | Intron 8 | 95.8 | 1 | 0.022 | 0.011 | 0.016 | 0.5476 | 5 |  |  |
| rs191389873 | C>T | Intron 8 | 94.7 | 1 | 0.034 | 0.033 | 0.033 | 0.9558 | 7 |  |  |
| rs576719453 | G>C | Intron 8 | 94.7 | 1 | 0.00 | 0.022 | 0.011 | 0.1643 | 7 |  |  |
| rs60540285 | A>T | Intron 8 | 95.8 | 0.8352 | 0.100 | 0.054 | 0.077 | 0.2479 | 6 |  |  |
| rs28515698 | T>C | Intron 8 | 67.4 | 1 | 0.083 | 0.029 | 0.055 | 0.1806 | 6 |  |  |
| rs4562992 | C>A | Intron 8 | 67.4 | 0.8612 | 0.28 | 0.294 | 0.289 | 0.8932 | 7 |  |  |
| rs28458188 | T>C | Intron 8 | 81.1 | 1 | 0.086 | 0.06 | 0.071 | 0.5297 | 7 |  |  |
| rs11400359 | ins1>del1 | Intron 8 | 97.9 | 1 | 0.100 | 0.073 | 0.086 | 0.5103 | 6 |  |  |
| rs191060835 | A>T | Intron 8 | 97.9 | 0.1072 | 0.033 | 0.021 | 0.027 | 0.5984 | 5 |  |  |
| rs868178022* | T>C | Intron 8 | 97.9 | 1 | 0.011 | 0 | 0.005 | 0.3004 | 5 |  |  |
| rs2053940 | G>A | Intron 8 | 100 | 1 | 0.106 | 0.073 | 0.089 | 0.4191 | 2b |  |  |
| rs185187566 | A>C | Intron 8 | 97.9 | 1 | 0.00 | 0.01 | 0.005 | 0.3316 | 4 |  |  |
| rs67688669 | T>C | Intron 8 | 100 | 0.5776 | 0.11 | 0.115 | 0.111 | 0.857 | 2b |  |  |
| rs188267947 | G>A | Intron 8 | 100 | 1 | 0.00 | 0.01 | 0.005 | 0.3211 | 2b |  |  |
| rs531891247 | A>T | Intron 8 | 100 | 1 | 0.011 | 0 | 0.005 | 0.3109 | 2b |  |  |
| rs150184166 | T>C | Intron 8 | 100 | 1 | 0.021 | 0.01 | 0.016 | 0.5483 | 2b |  |  |
| rs138823543 | T>C | Intron 8 | 100 | 1 | 0.011 | 0 | 0.005 | 0.3109 | 4 |  |  |
| rs1365771 | A>T | Intron 8 | 100 | 1 | 0.202 | 0.146 | 0.174 | 0.3058 | 4 |  |  |
| rs45512501 | T>G | Intron 8 | 100 | 1 | 0.096 | 0.062 | 0.079 | 0.3955 | 2b |  |  |
| rs6074 | C>A | Exon 9 | 100 | 1 | 0.085 | 0.062 | 0.074 | 0.551 | 3a |  |  |
| rs6075 | C>T | 3' flanking | 100 | 1 | 0.01 | 0.021 | 0.016 | 0.573 | 5 |  |  |
| [rs116817623](https://www.ncbi.nlm.nih.gov/projects/SNP/snp_ref.cgi?rs=116817623) | T>A | 3' flanking | 100 | 1 | 0.011 | 0 | 0.005 | 0.3109 | 5 |  |  |
| rs1978579 | C>T | 3' flanking | 100 | 1 | 0.106 | 0.073 | 0.089 | 0.4191 | 5 |  |  |
| rs113341022 | G>C | 3' flanking | 98.9 | 1 | 0.022 | 0.021 | 0.021 | 0.9657 | 3a |  |  |
| rs28427123 | T>C | 3' flanking | 98.9 | 1 | 0.065 | 0.062 | 0.064 | 0.9393 | 3a |  |  |
| rs112885895 | T>C | 3' flanking | 98.9 | 1 | 0.043 | 0.042 | 0.043 | 0.9509 | 3a |  |  |
| rs538960091 | G>A | 3' flanking | 98.9 | 1 | 0.011 | 0.01 | 0.011 | 0.9759 | 3a |  |  |
| rs1978578 | C>T | 3' flanking | 98.9 | 0.3421 | 0.446 | 0.427 | 0.436 | 0.7975 | 3a |  |  |
| HW-P: Hardy-Weinberg equilibrium p-value, MAF: Minor allele frequency, *Novel variants: Submitted to the dbSNP database as a first submission by handle ID: Kamboh  RegulomeDB scores were generated by using <http://regulome.stanford.edu/>. Scores represents; 1a- eQTL + TF binding + matched TF motif + matched DNase Footprint + DNase peak; 1b- eQTL + TF binding + any motif + DNase Footprint + DNase peak; 1c- eQTL + TF binding + matched TF motif + DNase peak; 1d- eQTL + TF binding + any motif + DNase peak; 1e- eQTL + TF binding + matched TF motif; 1f- eQTL + TF binding / DNase peak; 2a- TF binding + matched TF motif + matched DNase Footprint + DNase peak; 2b- TF binding + any motif + DNase Footprint + DNase peak; 2c- TF binding + matched TF motif + DNase peak; 3a- TF binding + any motif + DNase peak; 3b- TF binding + matched TF motif; 4- TF binding + DNase peak; 5-TF binding or DNase peak; 6-other. | | | | | | | | | | | |
