# Supplementary material for: Hepatic lipase (LIPC) sequencing in individuals with extremely high and low high-density lipoprotein cholesterol levels
Source: PLoS One. 2020 Dec 16;15(12):e0243919. doi: 10.1371/journal.pone.0243919 (PMC7743991; doi:10.1371/journal.pone.0243919)
Supplement: S4 Table — (DOCX) [file pone.0243919.s011.docx]

**S4 Table. Tagger results for 161 *LIPC* variants (MAF≥0.05, r^2^≥0.8) in ABs**

| **Bin** | **Test** | **Alleles Captured** | **Bin** | **Test** | **Alleles Captured** |
| --- | --- | --- | --- | --- | --- |
| 1 | rs4775079 | rs72062747,rs7180795,rs1869130,rs8030903,rs868805990,rs4774305,rs12438032,rs11071390,rs1839927,rs4775079,rs1839928,rs71425810,rs34964641,rs7181592,rs7170227,rs7181945,rs10851636,rs1869131 | 41 | rs78312967 | rs78312967 |
| 2 | rs6494022 | rs7169280,rs6494019,rs6494020,rs6494023,rs2414603,rs7168293,rs6494022,rs7176457,rs11637823,rs2414602,rs8038420,rs4775080,rs7163112,rs4775081,rs6494021 | 42 | rs2242062 | rs2242062 |
| 3 | rs28602186 | rs28427123,rs28458188,rs6074,rs28602186 | 43 | rs3751542 | rs3751542 |
| 4 | rs1973026 | rs1973025,rs1973026,rs4775077,rs1007542,rs1973027,rs1869146 | 44 | rs56143289 | rs56143289 |
| 5 | rs17301864 | rs17301864,rs397715435,rs56122478,rs36017602,rs17301857 | 45 | rs2242063 | rs2242063 |
| 6 | rs8027708 | rs8030893,rs1869132,rs10851637,rs8027708,rs4528512 | 46 | rs7165654 | rs7165654 |
| 7 | rs7173774 | rs7173774,rs11400359,rs1978579,rs2053940,rs60540285 | 47 | rs7175412 | rs7175412 |
| 8 | rs17190678 | rs41294813,rs17190678,rs56010348,rs67727760 | 48 | rs76588424 | rs76588424 |
| 9 | rs17269397 | rs10152558,rs17269397,rs67688669 | 49 | rs6078 | rs6078 |
| 10 | rs2227300 | rs73412742,rs2227300,rs41292504 | 50 | rs6494018 | rs6494018 |
| 11 | rs7181352 | rs7181352,rs1007543 | 51 | rs2233735 | rs2233735 |
| 12 | rs368318275 | rs73412744,rs368318275 | 52 | rs143731122 | rs143731122 |
| 13 | rs2414594 | rs2414596,rs2414593,rs2414595,rs2414594,rs61207362 | 53 | rs11858020 | rs11858020 |
| 14 | rs12593954 | rs12593954,rs12592139 | 54 | rs4572327 | rs4572327 |
| 15 | rs35412158 | rs35892254,rs35412158 | 55 | rs2242064 | rs2242064 |
| 16 | rs1973024 | rs1973023,rs1973024 | 56 | rs2233739 | rs2233739 |
| 17 | rs7175421 | rs1978578,rs7175421 | 57 | rs41292508 | rs41292508 |
| 18 | rs11639204 | rs8026372,rs4444272,rs2414600,rs7181367,rs2414601,rs7179938,rs2414599,rs11639204,rs28455962,rs7179940,rs2414598,rs7179747,rs7180130; | 58 | rs11631342 | rs11631342 |
| 19 | rs2414592 | rs2414592 | 59 | rs2414591 | rs2414591 |
| 20 | rs690 | rs690 | 60 | rs368337583 | rs368337583 |
| 21 | rs3829460 | rs3829460 | 61 | rs6076 | rs6076 |
| 22 | rs7166788 | rs7166788 | 62 | rs45512501 | rs45512501 |
| 23 | rs871804 | rs871804,rs28524122,rs12913969,rs12908645 | 63 | rs7171818 | rs7171818 |
| 24 | rs7171818.1 | rs7171818.1 | 64 | rs1365771 | rs1365771 |
| 25 | rs11632627 | rs11632627 | 65 | rs16940493 | rs16940493 |
| 26 | rs6084 | rs6084 | 66 | rs145482805 | rs145482805 |
| 27 | rs865899292 | rs865899292 | 67 | rs1869129 | rs1869129 |
| 28 | rs114971017 | rs114971017 | 68 | rs78268218 | rs78268218 |
| 29 | rs2242066 | rs2242066 | 69 | rs6082 | rs6082 |
| 30 | rs78861621 | rs78861621 | 70 | rs2070895 | rs2070895 |
| 31 | rs2414589 | rs2414589 | 71 | rs11071389 | rs11071389 |
| 32 | rs2233741 | rs2233741 | 72 | rs41294815 | rs41294815 |
| 33 | rs7176030 | rs7176030 | 73 | rs59699190 | rs59699190 |
| 34 | rs17190650 | rs17190650 | 74 | rs2242061 | rs2242061 |
| 35 | rs41292510 | rs41292510 | 75 | rs12592127 | rs12592127 |
| 36 | rs115464904 | rs115464904 | 76 | rs7178362 | rs7178362 |
| 37 | rs4775075 | rs4775075 | 77 | rs11633191 | rs11633191 |
| 38 | rs2242065 | rs2242065 | 78 | rs74017973 | rs74017973 |
| 39 | rs6083 | rs6083 |  |  |  |
| 40 | rs16940472 | rs16940472 |  |  |  |
