# Supplementary material for: Hepatic lipase (LIPC) sequencing in individuals with extremely high and low high-density lipoprotein cholesterol levels
Source: PLoS One. 2020 Dec 16;15(12):e0243919. doi: 10.1371/journal.pone.0243919 (PMC7743991; doi:10.1371/journal.pone.0243919)
Supplement: S5 Table — (DOCX) [file pone.0243919.s012.docx]

**S5 Table. Tagger results for the 78 *LIPC* variants (MAF≥0.05, r^2^≥0.8) in NHWs.**

| **Bin** | **Test** | **Alleles Captured** | **Bin** | **Test** | **Alleles Captured** |
| --- | --- | --- | --- | --- | --- |
| 1 | rs6074 | rs12908645,rs56122478,rs17301857,rs28602186,rs36017602,rs67262567,rs28427123,rs12913969,rs28524122,rs871804,rs2242063,rs2242066,rs17301864,rs6074 | 21 | rs12909325 | rs12909325 |
| 2 | rs41294813 | rs11858020,rs4528512,rs17190678,rs7166788,rs56010348,rs6084,rs41294813 | 22 | rs79783761 | rs79783761 |
| 3 | rs78312967 | rs77010273,rs2242065,rs12595265,rs6082,rs78312967,rs4775075 | 23 | rs17190650 | rs17190650 |
| 4 | rs8030893 | rs1869132,rs17269397,rs67688669,rs8027708,rs10851637,rs8030893 | 24 | rs2242064 | rs2242064 |
| 5 | rs11071389 | rs1973023,rs1973024,rs7165654,rs11071389 | 25 | rs56143289 | rs56143289 |
| 6 | rs7171818A>G | rs3751542,rs7175421,rs7171818A>G | 26 | rs6076 | rs6076 |
| 7 | rs74017973 | rs12592127,rs74017973 | 27 | rs33931419 | rs33931419 |
| 8 | rs7178362 | rs11633043,rs7178362 | 28 | rs11631482 | rs11631482 |
| 9 | rs55733523 | rs55733523,rs1973027 | 29 | rs7171818A>T | rs7171818A>T |
| 10 | rs11632627 | rs11632627,rs6494018 | 30 | rs2242062 | rs2242062 |
| 11 | rs16940472 | rs16940472 | 31 | rs59699190 | rs59699190 |
| 12 | rs11633191 | rs11633191 | 32 | rs2070895 | rs2070895 |
| 13 | rs143731122 | rs143731122 | 33 | rs7172821 | rs7172821 |
| 14 | rs11632970 | rs11632970 | 34 | rs6083 | rs6083 |
| 15 | rs8192701 | rs8192701 | 35 | rs11852861 | rs11852861 |
| 16 | rs690 | rs690 | 36 | rs2233741 | rs2233741 |
| 17 | rs2242061 | rs2242061 | 37 | rs72743035 | rs72743035 |
| 18 | rs45500398 | rs10459627,rs35892254,rs45500398 | 38 | rs2233739 | rs2233739 |
| 19 | rs1978578 | rs1978578 |  |  |  |
| 20 | rs7171818G>T | rs7171818G>T |  |  |  |
