# Supplementary material for: Hepatic lipase (LIPC) sequencing in individuals with extremely high and low high-density lipoprotein cholesterol levels
Source: PLoS One. 2020 Dec 16;15(12):e0243919. doi: 10.1371/journal.pone.0243919 (PMC7743991; doi:10.1371/journal.pone.0243919)
Supplement: Attachment — Submitted filename: Response to Reviewers.docx [file pone.0243919.s013.docx]

**Manuscript no:** PONE-D-20-29487

Hepatic Lipase (*LIPC*) sequencing in individuals with extremely high and low high density lipoprotein cholesterol levels

Dear Editors:

Thank you for the opportunity to revise our manuscript. We are pleased with the overall positive review and found the comments very helpful in improving our manuscript. We have revised the manuscript accordingly (highlighted in red in the manuscript) and below we provide a point-by-point response to each comment in red.

The Funding Statement has been also updated as "This study was supported by the National Heart, Lung and Blood Institute (NHLBI) grant, HL084613 (M. Ilyas Kamboh). The funders had no role in study design, data collection and analysis, decision to publish, or preparation of the manuscript. There was no additional external funding received for this study ".

**Reviewer #1:**

In this study, Pirim D and colleagues performed a resequencing of the LIPC gene in 95 non-Hispanic Whites (NHWs) and 95 African blacks (ABs) selected from the upper and lower 10th percentile of HDL cholesterol distribution. The aim of the study was to identify and understand the role of common, uncommon and rare variants that may influence plasma HDL-C levels. They identified a total of 464 variants, including 43 novel. To assess their functional relevance, in silico functional analyses were executed. Two nonsynonymous variants (p.S289F, p.T405M), found in NHWs with higher HDL-C levels were predicted to have damaging effect on LIPC protein. Furthermore, the authors found several non-coding variants that possibly reside in the circRNA and lncRNA binding sites and may have regulatory function. The authors conclude that this study highlights the importance of LIPC polymorphisms, common and rare, in influencing plasma HDL-C levels and that functional studies are needed to further evaluate the roles of non-coding regulatory variants in the lipid metabolism.

The results presented in the paper are well written and easy to follow and provide additional evidences on the importance of the LIPC gene polymorphisms in the modulation of plasma HDL-C levels.
Nonetheless, I would have some comments to the authors.

**Response:** We greatly appreciate the reviewer for noting that our manuscript is well written, easy to follow and the importance of novel findings.

1) It’s not clear to me why the authors state that they identified a total of 464 variants if summing the 122 shared in both populations, the 156 unique to NHWs and the 234 unique to Abs, the variants are 512 in total.

**Response:** We apologize for this typo as the number of variants unique to NHWs should have been 56 not "156", so the total number of identified variants is 412. Abstract, results and discussion sections have now been edited accordingly.

2) I found a discrepancy between S2 Table where are reported 180 variants in NHWs of which 88 are common and the test in page 8 “Of the 178 variant identified in NHWs, 86 were common (MAF≥0.05)….”. In paragraph 3.2 (line 4) the authors state they found 88 common variants.

And the same for S3 Table: 358 variants of which 174 common in AB cohort and in the test on page 8 “Among the 356 variants found in ABs, 172 were common (MAF≥0.05)……”
Could the authors explain the differences?

**Response:** The discrepancy was due to a triallelic variant (rs7171818) that was found in both populations. Including the triallelic variant, the total number of common variants identified in NHWs and ABs were 86 and 172, respectively. However, since the three alleles for the rs7171818 were presented in 3 separate rows, the number of total variants falsely got added to 88 and 174 in NHWs and ABs, respectively. In order to avoid this apparent mis-representation, now the triallelic variant in Table S2 and S3 is presented in a single row.

Of note, Haploview analyses was conducted for all variants, including three alleles for the triallelic variant. For further clarification, we have edited the text on Page 8, Section "TagSNP identification and Linkage Disequilibrium (LD) analyses", line 2-6 as follows:

“Of the 174 common variants in ABs (including 3 alleles of the triallelic variant rs7171818, See S3), 3 deviated from Hardy-Weinberg equilibrium (HW-P-value<0.0001) and 10 had low call rate (<80%). Of the 88 common variants in NHWs (including 3 alleles of the triallelic variant rs7171818, See S2), 9 had low call rate (<80%) and 1 deviated from Hardy-Weinberg equilibrium (HW-P-value<0.0003).”

3) in paragraph 3.2, it’s not clear why 161 variants in AB cohort were analysed (S4 Table) if the common variants were 172. I wonder if the 3 variants deviating from H-W equilibrium and the 10 with low call rate have been eliminated. However, in this case the variants analysed in S4 Table should be 159. This should be clarified.

**Response:** In ABs, 174 common variants (including the triallelic variant rs7171818) were analyzed. "172" was a typo, which is now corrected on Page 8, Section "TagSNP identification and Linkage Disequilibrium (LD) analyses", line 2-4, as indicated above.

4) I’ve a doubt how the authors calculated the RegulomeDB Score in Table 3. Indeed, I tried to calculate the Score selecting randomly few snps. However, the results are different from those reported in table 3. May the authors explain these different results? I wonder if the RegulomeDB scores calculated in S2 and S3 Tables present the same discrepancy.
NHWs
RefSNP ID RegulomeDB rank
rs117911817 5
rs1869129 3a
rs1869130 4
rs12438032 5
rs34964641 5
rs35925692 5
rs1869131 5
rs4775079 5

ABs
RefSNP ID RegulomeDB rank
rs35631005 4
rs533300601 3a
rs16940468 5
rs12909325 5
rs115408618 3a
rs190375050 5
rs181084356 5
rs568646677 4
rs143889538 2b

**Response:** Thank you for pointing this out. We used the RegulomeDB version 1 in our analyses and since then the database has been updated, resulting in difference scores for some variants than the previous version.

We have now reanalyzed all variants by using RegulomeDB v2 and updated all scores in the tables and related text accordingly.

**Reviewer #2:**

Pirim and collaborator sequenced LIPC gene in 190 subjects selected from the upper and lower 10th percentile of HDL levels in two cohorts, African Blacks and non-Hispanic Whites. 464 variants were recorded, 43 of which were novel. SNPs were evaluated using some predictive online software for coding and non-coding (regulatory) variants. Among all, 17 coding variants were identified: 6 cause aminoacid change while 11 were synonymous. Authors analysis were focused on difference in MAF distribution within the extremes of HDL levels. Overall, 3 variants showed a significant different distribution. In particularly, MAF were lower in low-HDL than high-HDL group. Moreover, authors found 3 common variants associated with extremes of HDL levels. Other findings are in line with previous published studies.

Language and exposition are very clear. Also the amount of data, figures, tables and supplementary materials provided make easy to follow authors argument. Interestingly, the analysis considers cohorts from different ethnic group, non-Hispanic White and African Black.

**Response:** We greatly appreciate the reviewer for noting multiple strengths in our study.

**Minor revision:**

Lacks of calculation (even if cited at page 15). As it would be useful for readers and for fully understand study design, could authors add a power calculation in statistical analysis paragraph, 2.4?

**Response:** We have now calculated the power by using G power based on our sample size and the text has been revised as below on Page 6, Section "Statistical and Bioinformatic Analysis", line 4-6:

“G power software (version 3.1) was used for power analysis, which showed that our sample size has sufficient power (97% at 5% alpha level) to detect low frequency variants (MAF<0.05) with moderate effect sizes (Cohen's d=0.05).”

Conclusion paragraphs seems too short. Could authors add a brief summary of findings, or most promising variants, or some hypothesis for observed effects in coding and regulatory variants, or some future prospective of the study?

**Response:** Since the summary of the findings is already highlighted in the Abstract and the promising variants discussed in the Discussion section, we avoided their repetitions in the Conclusions. However, in deference to the reviewer's suggestion, we have added the the below text in the Conclusions section on Page 16, line 3-6:

“Our results also emphasize the possible regulatory impact of non-coding *LIPC* variants in the determination of HDL-C levels. However, associations between suggested regulatory *LIPC* variants and lipid traits should be tested in large samples....”

at page 8. On paragraph 3.1 the common variants found in NWHs are n=86, while in the same page, but in paragraph 3.2, are n=88. Please correct or explain.

**Response:** As responded to under Reviewer 1, in NHWs, we identified 86 common variants, including one triallelic SNP (rs7171818) and for clarification, we have edited the text on Page 8, Section "TagSNP identification and Linkage Disequilibrium (LD) analyses", line 2-6.

We hope that we have satisfactorily addressed reviewers’ concerns and look forward to learning a favorable response.

Sincerely,

M. Ilyas Kamboh, PhD, FAHA

On behalf of all authors
